# Theoretical Framework for Facilitating Young Musicians’ Learning of Expressive Performance

**DOI:** 10.3389/fpsyg.2020.584171

**Published:** 2021-01-11

**Authors:** Henrique Meissner

**Affiliations:** Department of Music, The University of Sheffield, Sheffield, United Kingdom

**Keywords:** aural modeling, dialogic teaching, embodied learning, expressivity, instrumental music teaching and learning, music performance expression, performance pedagogy, teaching children expressiveness

## Abstract

Since communication and expression are central aspects of music performance it is important to develop a systematic pedagogy of teaching children and teenagers expressiveness. Although research has been growing in this area a comprehensive literature review that unifies the different approaches to teaching young musicians expressiveness has been lacking. Therefore, the aim of this article is to provide an overview of literature related to teaching and learning of expressiveness from music psychology and music education research in order to build a new theoretical framework for teaching and learning expressive music performance in instrumental music lessons with children and teenagers. The article will start with a brief discussion of interpretation and expression in music performance, before providing an overview of studies that investigated teaching and learning of performance expression in instrumental music education with adults and children. On the foundation of this research a theoretical framework for dialogic teaching and learning of expressive music performance will be proposed and the rationale explained. Dialogic teaching can be useful for scaffolding young musicians’ learning of expressivity as open questions can stimulate thinking about the interpretation and may serve to connect musical ideas to the embodied experience of the learner. A “toolkit” for teaching and learning of expressiveness will be presented for practical application in music lessons. In addition, a theoretical model will be proposed to further our understanding of teaching and learning of expressive music performance as a multifaceted and interactive process that is embedded in the context of tutors’ and learners’ experiences and environment. Finally, implications of this framework and suggestions for future research will be discussed.

## Introduction

Performing music expressively is an essential element of music participation as expressivity enhances the playing and listening experience. A growing body of literature is addressing the need for research of effective methods for facilitating children’s learning of expressiveness^1^ as a systematic approach has been lacking (Lisboa, 2008; McPhee, 2011; cf., Brenner and Strand, 2013). A literature review synthesizing several approaches to facilitating young musicians’ learning of expressiveness can enable researchers and teachers to develop a systematic pedagogy in this area thus enhancing instrumental^2^ teaching practice. What can we^3^ learn from research findings in this area, and how might this enhance our teaching practices?

It is important to develop this area of teaching and learning as there are indications in the literature that there is limited attention for expression and communication in lessons when children begin formal instrumental music learning (Rostvall and West, 2003, 2005; West and Rostvall, 2003; Karlsson and Juslin, 2008; McPherson et al., 2012). In a longitudinal study in which 157 young musicians were followed from their seventh to their 20-s birthday, McPherson et al. (2012) reported that they did not find evidence of teaching aimed at musical communication or expression:

It is difficult to find any data in our study that suggest that the students were explicitly learning about the communicative and expressive potential of playing those notes during the early years of learning. (McPherson et al., 2012, p. 219).

The authors found that musical participation for most students in their sample focused on the technical side of playing, which was not helpful for the development of a meaningful interaction with music. Some have suggested that the reason for limited instruction in performance expression might be that knowledge of expressivity is often tacit and intuitive (Lindström et al., 2003; Juslin et al., 2004). Consequently, it might be difficult to explain to others *how* to perform expressively. As it is important to understand how instrumental tutors can enhance expression and communication in learners as they are performing and creating music (cf., Hallam, 2010, p. 809), this article aims to develop a theoretical framework for teaching and learning expressive music performance by exploring the following research questions: (1) How can instrumental music teachers facilitate young musicians’ learning of expressive music performance; Which methods are effective for teaching expressiveness? (2) What do children find helpful for their learning of expressive music performance?

Firstly, musical interpretation and performance expression will be discussed and secondly research findings on teaching and learning expressive music performance (EMP) will be reviewed. Next, a theoretical framework for teaching and learning expressive performance with young musicians will be proposed. The aim of this framework is to outline how instrumental and vocal tutors can guide and facilitate young musicians’ learning of expressive performance of works of Western classical music. A practical “toolkit” for teaching and learning of expressivity will be presented and a theoretical model will illustrate how teaching-and-learning of EMP is a multifaceted and interactive process. For this work, an expressive music performance is defined as a performance in which the musician conveys their interpretation of the *compositional structure* and *musical character* convincingly to a listener (Meissner, 2018; Meissner and Timmers, 2020). *Musical character* refers to the affects, atmosphere, emotions^4^, ideas, imagery, or motions that may be associated with a musical work (Shaffer, 1992, 1995; Brendel, 2011).

## Background

### Music and Interpretation

The theory and practice of teaching and learning expressiveness is influenced by our understanding of music, interpretation and performance expression (Swanwick, 2011; Doǧantan-Dack, 2014; Bonastre et al., 2017). Therefore, it is important to explore our understanding of these concepts and to be aware that these are difficult to define (cf., Doǧantan-Dack, 2014; Epperson, 2016). Music can be described as “that form of interhuman communication in which humanly organized non-verbal sound can, following culturally specific conventions, carry meaning relating to emotional, gestural, tactile, kinetic, spatial and prosodic patterns of cognition” (Tagg, 2012, p. 44)^5^. Although the notation that is traditionally used for Western art music contains a representation of rhythm and pitch, most musicians will agree that it cannot represent all the intuitive aspects required for an expressive performance (e.g., Howat, 1995; Palmer, 1997; Gibbs, 2015) conveying all the composer’s intentions “let alone the full range of meanings a work can express” (Silverman, 2007, p. 103). According to Silverman (2007) performers, teachers and scholars hold a variety of interpretive perspectives ranging from a *formalist* view to a *subjective* view of performance. In a formalist view, musicians aim to let the score “speak for itself”; the performer “only sounds out a notated score in strict accordance with a composer’s instructions” (p. 102). In contrast, according to a subjective view, performers have the freedom to interpret and realize a score based on their own ideas and feelings. Between these two opposite positions various viewpoints are possible. Based on Rosenblatt’s (1938) transactional theory, Silverman makes a distinction between an *efferent* and an *aesthetic* reading of a score. In an efferent reading the reader-performer reads the score analytically, to learn which pitch, rhythms and dynamics should be played, while an aesthetic reading includes reflection and artistic meaning making. Silverman proposed that “the sonic event we call music is (…) an event in time; it is constructed and experienced in the mind of listeners or performers when they ‘meet’ a notated score” (Silverman, 2007, p. 107). According to Silverman the performer of a score, and/or the listener together with the mental or actual sounds, create musical meaning^6^. The listener/performer’s interpretation of the music may vary over time depending on experiences and environment. Likewise, Doǧantan-Dack (2014) proposed that “the music” is not in the notated score, but “comes into being only in the act of music making” (p. 12). Thus, musical meaning comes into existence when it is performed and heard and varies depending on the performer and situation. Therefore, musicians need to reflect on their interpretation of the meaning of a musical work:

Interpretation is the act of bringing one’s whole being – intellectual, social, cultural, artistic, physical, emotional, and personal – into the performing event. If this is not done, the result is nothing more than a production; it is merely an aural photocopy of a score. Conversely, without careful attention and study of a score, a performer will offer little more than pure subjectivity. Artistic-aesthetic interpreting depends on the continuous interplay of efferent and aesthetic processes. (Silverman, 2007, p. 109).

Consequently, it seems likely that the meaning that is expressed by a performer is different to the composer’s intention or the listener’s perception (see Shaffer, 1992, p. 265), as the composer’s intentions, the performer’s interpretation and listener’s perception of the musical meaning are embedded into the contexts of their surrounding worlds (cf., Silverman, 2020) and affected by their experience, expectation, mood, personality, preference, situation and environment (Gabrielsson, 2001; Cross, 2005; Silverman, 2007; Honing, 2009; Tagg, 2012; Doǧantan-Dack, 2014; Ashley, 2017; Cespedes-Guevara and Eerola, 2018). Therefore, there cannot by one “prototypical” or “model” performance of a composition (cf. Timmers and Honing, 2002; Fabian et al., 2014) as there can be various appropriate interpretations within stylistic constraints. Silverman’s transactional theory of musical interpretation is important for music education as it implies that educators should teach students to reflect on and develop their interpretation of a musical work which can vary depending on the performer and situation (cf., Trapkus, 2020).

### Expressive Music Performance

Empirical research has demonstrated that expert musicians use various expressive tools such as articulation, dynamics, tempo, timing, timbre, attack, decay, intonation, vibrato, ornamentation, and ancillary gestures to convey their interpretation of a musical work to their audience (e.g., Juslin and Persson, 2002; Juslin, 2003; Fabian et al., 2014). Generally, three main conceptions of EMP can be found in music psychology research: firstly, some emphasize the importance of conveying musical structure convincingly in performance expression (e.g., Seashore, 1923; Clarke, 1988; Palmer, 1997; Friberg and Battel, 2002; Juslin, 2003). This view is related to a formalist theory of musical meaning in which the structure of a composition is seen as the most important feature for understanding a musical work (see DeBellis, 2005). Musicians can manipulate various performance features, e.g., articulation, dynamics, tempo, gestures (MacRitchie et al., 2013) and “ensemble timing” (Friberg and Battel, 2002) to communicate phrases and harmonic structures (e.g., Todd, 1985; Repp, 1992, 1998; Palmer, 199). Secondly, many scholars subscribe to a referentialist conception of musical meaning, perceiving music as resembling or expressing extra-musical features, such as affects (Cespedes-Guevara and Eerola, 2018), emotions, moods, feelings, motion, characters, or patterns of sound (Gabrielsson, 1999; Gabrielsson and Juslin, 1996; Juslin and Laukka, 2000; Juslin and Persson, 2002; Juslin, 2003; DeBellis, 2005; Brendel, 2011). Shaffer (1992) suggested that, for understanding performance expression and developing an interpretation, it might be helpful to think of music as conveying an “abstract narrative” containing a protagonist with a “musical character.” Performers can invent a musical character in response to moods and structures perceived in the music to “solve a series of expressive problems” and create an expressive performance (Shaffer, 1995, p. 31). Likewise, Watt and Ash (1998) proposed that music might be perceived as if it were a virtual person making a disclosure. To convey the musical character, performers may modulate various expressive devices, such as articulation, dynamics, tempo, timing, timbre, attack, decay (Juslin and Persson, 2002; Juslin, 2003; Juslin and Timmers, 2010), intonation (Johnson, 2004), vibrato (Timmers and Desain, 2000; Juslin et al., 2004), ornamentation (Windsor et al., 2000; Timmers and Ashley, 2007) and ancillary gestures (Davidson, 1993, 2005; Davidson and Correia, 2002; Wanderley and Vines, 2006; Dahl and Friberg, 2007). Especially expressive tools such as dynamics, mode, harmony, pitch, tempo and rhythm can shape the perception of emotion in music (Gabrielsson and Lindström, 2010), while melodic contour and pitch can contribute to this too (Schellenberg et al., 2000; Coutinho and Dibben, 2013). However, Cespedes-Guevara and Eerola (2018) proposed that music communicates core dimensions of affects (arousal and valence) rather than basic emotions. They argue that “the phenomenon of perception of emotions in music arises from the interaction of music’s ability to express core affects and the influence of top-down and contextual information in the listener’s mind” (Cespedes-Guevara and Eerola, 2018, p. 1). Although this hypothesis sounds plausible, this idea is not practical for a pedagogy of teaching and learning expressiveness, as it seems likely that tutors and learners need metaphors referring to emotions, moods or character for developing their understanding and interpretation of the works they study. An overview of music and emotion research can be found in Timmers (2018).

Thirdly, Nusseck and Wanderley (2009) proposed that EMP is characterized by *expressive intensity* and *musical tension.* Their *expressive intensity* is related to the degree of expressiveness of the performance (Timmers et al., 2006; Fabian et al., 2014) and is similar to Schubert and Fabian’s (2014) *expressiveness amount* and Davidson’s (1993) *performance manner*. Nusseck and Wanderley describe high *musical tension* as a “feeling of excitement and involvement, whereas low tension refers to uncertainty and unsteadiness” (p. 338). Also related to musical tension is a sense of *forward movement* or *forward energy* (cf. Broomhead, 2006): Musicians can experience a sense of *forward movement* whilst playing; metaphorically speaking, the music has direction and “flows” toward the end of a phrase, cadence or “tipping point” (Chew, 2013, 2014). In contrast, when a performance is not going well, players have the impression that the music is static; the performance feels heavy and performers and listeners might lose focus. It is common knowledge among music teachers that compositions requiring a slow tempo are harder to perform expressively than fast and virtuosic works, because they demand more *musical tension* and more focus on *forward movement* to be perceived as expressive (Meissner, 2018). It seems likely that *musical tension* and *forward movement* contribute to the perceived *expressive intensity* of a performance.

Furthermore, Schubert and Fabian (2014) proposed that *stylishness* is an important feature of EMP. *Stylishness* refers to expressiveness appropriate to performance conventions (Schubert and Fabian, 2014, referring to Kendall and Carterette, 1990; Fabian and Schubert, 2009), for example Baroque music played with historically informed ornamentation, articulation and expressiveness. Finally, the expressiveness of a performance is influenced by the interaction of the musician with the audience (e.g., Doǧantan-Dack, 2014). In every music performance there is a listener present, even in the rehearsal room. During practice musicians communicate with themselves, in ensemble rehearsals musicians communicate with each other and in lessons teachers and students listen to the music that is performed (Tagg, 2012; Doǧantan-Dack, 2014). In each situation the real or imaginary audience affects the performance.

#### Performance Expression Models

Some scholars proposed theoretical models representing various aspects of performance expression to provide a foundation for teaching or research in this area.

##### GERMS model

The GERMS model of performance expression by Juslin (2003) describes five aspects of expressive performance: *Generative rules; Emotional expression; Random fluctuations; Motion principles*; and *Stylistic unexpectedness*. *Generative rules* refer to the communication of a composition’s structure. The musician’s interpretation of the musical structure generates expressive variations that communicate the phrases and sections of a composition (e.g., Clarke, 1988; Palmer, 1996) as described above. *Emotional expression* refers to the communication of emotion in performance through various expressive cues as explained above. According to Juslin emotional expression has a strong effect on listeners’ evaluation of expressiveness and is an important aspect of performance expression (Juslin, 2003; Lindström et al., 2003; Juslin et al., 2004). The use of expressive cues varies among performers, instruments and styles (Juslin, 2000; Juslin and Timmers, 2010).

To describe the process of emotional communication in music performance Juslin proposed a modified *Brunswik lens model* (Juslin, 2000; Juslin and Laukka, 2003; Juslin and Timmers, 2010). This model shows how musicians convey emotions by modifying various uncertain and partly redundant expressive cues such as tempo, loudness, timbre and articulation, which can be recognized and decoded by listeners. Timmers (2020) suggested that the perception of emotion in music is not only a function of the values of certain musical cues but also a function of the probability of a particular emotion in musical contexts and the uniqueness of the cue to differentiate emotion.

*Motion principles* refer to tempo changes that reflect naturally occurring movements from animate or inanimate origin (Lehmann et al., 2007). For example, final ritardandi in music performances tend to follow a similar pattern to runners’ decelerations (Friberg and Sundberg, 1999). Although music can express emotion or movement, a composition could also be expressive of other ideas or imagery as mentioned above.

*Random fluctuations* are a result of the limitations in motor precision and contribute to the “living” character of the music (Juslin, 2003), while *stylistic unexpectedness* comprises “local deviations from performance conventions” (Juslin, 2003, p. 273); Musicians often diverge from stylistic conventions to create a unique and idiosyncratic performance (Lehmann et al., 2007). Juslin acknowledged that musicians generally aim to limit random fluctuations, while many seek to increase the originality of their interpretation and performance (Juslin, 2003, p. 287).

Overall, Juslin’s GERMS model explains how music performance can be expressive of musical structure, emotions or movement, whilst stylistic unexpectedness and random fluctuations also shape listeners’ perception of expressiveness. This model can be helpful for thinking about teaching performance expression, as it explains how this phenomenon consists of various components that can be taught and practiced. However, this model does not yet account for the effects of expressive intensity or stylishness. Not all forms and degrees of stylistic unexpectedness are valued as contributing positively to the overall expressiveness of a performance (Schubert and Fabian, 2014). It depends on the enculturation, personality, and preference of the performer, teacher and/or listener to what extent such stylistic unexpectedness is acceptable (Juslin, 2003; Schubert and Fabian, 2014; Meissner, 2017).

##### Taxonomy of performance expression

Stylishness and expressive intensity are accounted for in Schubert and Fabian’s (2014) taxonomy of performance expression. This model distinguishes two types of *expression content*, namely musical expressiveness and emotional expressiveness. Furthermore, this taxonomy consists of two *expression layers*, a compositional and a performance layer, both comprising of musical and emotional expression content. The compositional layer is based on the “structural elements of music that are determined by a musical score/composer” (ibid., p. 286) whilst the performance layer relates to the manipulation of expressive devices such as articulation, tempo and dynamics by the musician. Thus, the compositional layer consists of musical content, the “objective expressiveness” (Schubert and Fabian, 2014, referring to Gilman, 1892a,b) of the composition, and emotional content which is related to “emotional correlates” of compositional features (ibid., p. 287, referring to Gabrielsson and Lindström, 2010) which may include other forms of expressive content too, like imagery or motion. The performance layer comprises two salient dimensions of musical expression content, namely *stylishness* and *expressiveness amount*. As mentioned above, stylishness refers to expressiveness appropriate to performance conventions while expressiveness amount relates to the expressive intensity of the performance. Thus, the performer’s manipulation of expressive devices to convey emotion refers to the emotional expressiveness content of the performance layer. However, the question arises whether it is feasible to make a distinction between musical and emotional expression content of a performance or a composition, as it seems likely that aspects that could be labeled as musical expressiveness content contribute to emotional expressiveness content too (cf., Schubert and Fabian, 2014).

#### Summary

In summary, music can express, refer to, or symbolize, non-musical concepts such as affects, atmosphere, characters, emotions, feelings, moods, movement and ideas and may induce an affective experience in performers and/or listeners (Doǧantan-Dack, 2014). Reflecting on and conveying one’s interpretation of the character and structure of a work are central aspects of performance expression, as well as *stylishness, expressive intensity, musical tension*, and *forward movement.* All these features contribute to the *expressiveness* of a performance. Juslin’s GERMS model as well as Schubert and Fabian’s taxonomy are helpful frameworks for understanding performance expression as a multifaceted phenomenon. However, these models do not yet explain how teachers can teach, or how learners might learn expressive performance. It is important to consider how theory related to performance expression can be developed to make it operational for performance pedagogy contexts.

For young musicians’ learning of EMP, it seems likely that instruction related to the interpretation and communication of *musical character* and *structure* can be effective. Consequently, instructional strategies and music education research should focus on these aspects of expressivity. It seems possible to discuss and explore phrasing of a composition with a child or teenager and to ask questions about the perceived affect or musical character. In contrast, it does not seem practical to discuss the *expressive intensity* or *musical tension* with a young child, as these concepts might be difficult to comprehend for children under 10. Although music does not necessarily communicate emotions, it can be helpful to describe music’s expressive character with metaphors referring to emotions, feelings, imagery or ideas during the learning process (see Langer, 1957; Leech-Wilkinson and Prior, 2014; Meissner and Timmers, 2020).

### Methods for Teaching Expressive Music Performance

Music psychology research has demonstrated that *verbal teaching using metaphors*, *verbal teaching explaining musical properties* and *aural modeling* (Woody, 2000; Woody, 2006a) can be effective methods^7^ for improving expressiveness in tertiary students and adult musicians. *Verbal teaching using metaphor* employs imagery to illustrate what the music should portray or sound like (e.g., Barten, 1992). Instruction using metaphors for enhancing expressiveness seems to be used extensively by conservatoire teachers (Lindström et al., 2003) and in various musical styles (Schippers, 2006). Metaphors are culturally defined (Schippers, 2006) and can be used in a variety of ways; for example to illustrate the musical character, for explaining technical instruction (Woody, 2006a) or to induce feelings in the performer (Woody, 2000, 2006a; Lindström et al., 2003; Schippers, 2006). It seems that some musicians translate metaphors into expressive musical tools via cognitive processes, while others personalize the imagery used to enhance their performance (Woody, 2006b). Although metaphors can be useful for explaining concepts that are difficult to describe verbally (e.g., Schippers, 2006) they can also be problematic when the imagery used is vague, complex or obscure (Persson, 1996; Schippers, 2006).

*Verbal teaching explaining concrete musical properties* describes how expressive tools such as variations in articulation, dynamics, and tempo can be employed to produce certain effects. Woody (2006a) found that instruction based on this type of teaching showed good consistency in affecting change leading to more expert performance. However, it required significantly more practice time than instruction using metaphors or aural modeling.

*Aural modeling* is widely used (Woody, 2000; Lindström et al., 2003) and can be described as listening to expert performances from professional musicians, life or via recordings, in order to build up internal aural representations of the music (Hallam, 1998) and to develop an expressive style (Sloboda, 2005; Meissner, 2017). Woody (2003) found that musicians who could describe verbally what they had heard in a musical model were more likely to reproduce it correctly than performers who were unable to give a verbal description. This effect was stronger for “non-idiomatic” features than for features typical for the genre. This suggests that describing expressive variations verbally might be a useful learning tool. Woody concludes that it is important to combine aural modeling with verbal communication (Woody, 2003). Although some researchers initially proposed that aural modeling might be less effective than verbal teaching because there is a considerable amount of detailed information contained in an aural model (Sloboda and Davidson, 1996; Woody, 2000) research has shown that this is not the case. Woody found that all three instructional strategies, verbal teaching using metaphors, verbal teaching explaining musical properties and aural modeling, were effective for improving expressivity (Woody, 2006a).

In contrast, Rosenthal (1984) found that aural modeling was more effective than concrete verbal instruction, or modeling combined with verbal teaching. The inconsistency between these findings might be caused by the differing levels of complexity of the test extracts and the content of the verbal instructions. Woody used short, simple extracts and the concrete verbal instructions as well as the metaphors were clear and focused on a few performance aspects, for instance articulation, dynamics, and some tempo changes. Conversely, in Rosenthal’s study a relatively complex test piece was used with many detailed instructions asking the performers to focus on tempo and style, rhythmic interpretations, phrasing, and dynamic markings. It seems possible that participants in Rosenthal’s study had to concentrate on too many performance cues which might have hindered their expressiveness.

Van Zijl and Sloboda (2011) and Van Zijl et al. (2014) explored the effect of musicians’ *experienced emotions* on performance characteristics. Eight violinists were asked to play a musical phrase that was expressive of sadness in response to three different instructions: (1) playing while focusing on technique; (2) giving an expressive performance; and (3) playing while focusing on experienced emotions after a mood induction procedure. Experienced emotions were defined as “music-related felt or induced emotions of the performer, as opposed to practice- or performance related emotions, or perceived emotions” (Van Zijl et al., 2014, p. 35, referring to Gabrielsson, 2001; Van Zijl and Sloboda, 2011). The violinists performed the melody in each performance condition. Findings revealed that a focus on technique produced a technically controlled performance without much personal expression. Concentrating on expressiveness resulted in an extraverted and “projected”, audience-directed performance, while a focus on experienced emotions created “more introverted and personal performances” (Van Zijl et al., 2014, p. 33). Six out of the eight violinists in this sample thought that this “emotional” performance was their best. As mentioned by the authors, these findings concern the performance of music expressive of sadness, and it is possible that these treatment conditions have different effects for extracts portraying other musical characters. As all participants participated in all three treatment conditions, it is conceivable that the order and practice influenced the research outcome too (Van Zijl et al., 2014). A similar study explored listeners’ preferences and evaluations of performances generated in these three conditions and found that listeners preferred the audience-directed performances and rated these as more “skilled” than the “technical” or “emotional” performances. The “emotional” performances were rated as more expressive of sadness than audience-directed or technical performances (Van Zijl and Luck, 2013).

Furthermore, constructive *feedback* can be useful for improving expressivity (Woody, 2003; Lehmann et al., 2007). Tutors can give constructive verbal feedback, addressing technical and musical issues (Hallam, 1998). Additionally, audio or video recordings of students’ performances can be used for obtaining feedback (e.g., Woody, 2000, 2001; Juslin et al., 2004), or musicians can receive feedback from technology (Juslin et al., 2004; Timmers and Sadakata, 2014). Although research has demonstrated that it is possible to improve performers’ emotional expression via computer software (Juslin et al., 2006), musicians seem reluctant to adopt this technology (Karlsson et al., 2009) as they prefer actual tutors explaining strategies for enhancing expressiveness (Karlsson et al., 2009; Timmers and Sadakata, 2014). Additionally, the use of computer-assisted feedback may be seen as cumbersome or time consuming (Timmers and Sadakata, 2014).

To summarize, verbal teaching using metaphors, concrete verbal teaching explaining musical properties, aural modeling, focusing on experienced or felt emotion, and constructive feedback can be effective methods for developing musicians’ expressiveness. Most of the participants in the abovementioned studies were tertiary students and/or advanced musicians who have persevered in their music studies after several years of musical participation. It seems likely that learning of expressive performance skills by young musicians is affected by their age (cf. Bonastre et al., 2017; Bonastre and Timmers, 2019), development and ability (Woody, 2006b; Meissner, 2017). Therefore, it is important to investigate which instructional strategies can be effective for children’s learning of EMP.

### Methods for Facilitating Children’s Learning of Expressive Music Performance

Since research has demonstrated that infants have innate gifts for musical communication (Honing, 2012, 2018; Tan et al., 2014; Háden et al., 2015; McPherson and Hallam, 2016), it should be possible for most children to develop their expressiveness in performance. Infants have a great interest in music (e.g., Trehub, 2016) and listen attentively to infant-directed speech and song (Trevarthen, 2002) in which parents convey affective intentions, modifying infant behavior and arousal levels (Trehub and Nakata, 2001; Trehub et al., 2010; Trehub, 2016). Young children tend to display expressive vocalizations and enjoy exploring musical sounds (Moorhead and Pond, 1942; Moog, 1976; Trehub and Nakata, 2001; Tafuri, 2008; Addessi, 2009; Delalande and Cornara, 2010). It seems likely that children’s experiences with infant-directed speech and song, vocalizations and musical play form the foundation for communication and expression in music performance when children grow up and start formal instrumental lessons (cf., Sloboda and Davidson, 1996; Trehub, 2003; Sloboda, 2005; Juslin and Timmers, 2010).

#### Observational Studies Investigating Young Musicians’ Learning of Expressiveness

Systematic empirical research into teaching children expressive performance started at the beginning of this century after some observational studies found that music lessons tend to focus on reading from notation and technique (Rostvall and West, 2003, 2005; West and Rostvall, 2003; Karlsson and Juslin, 2008). Three studies used observation of choir and instrumental lessons for investigating teaching and learning of expressiveness with young musicians (Table 1). Brenner and Strand (2013) observed lessons of four tutors and found that aural modeling, verbal instructions and task repetitions were used for improving children’s (aged 7–15) EMP. Brenner and Strand observed that tutors adjusted their teaching to children by exaggerating dynamics and melodic contour of verbal instructions, enlarging gestures accompanying verbal explanations, and overdoing aural modeling. Modeling was used consistently and as main strategy. Although tutors in this study thought that emotion is important for musical expression they varied “on ideas about how and why to focus upon emotions and physical sensations during lessons as well as how to go about drawing these out from the students” (Brenner and Strand, 2013, p. 92). It seems therefore that the teachers were not sure how to elicit EMP in their pupils, although they were convinced that working on emotion in music is important for teaching expressivity.

**TABLE 1:** Overview of empirical studies investigating instructional strategies for facilitating young musicians’ (aged 5–16) learning of expressive performance.

Source	Type of study	Instructional strategies	Participants	Outcome
Brenner and Strand, 2013	Case studies: interviews and lesson observation	Modeling, verbal teaching consisting of analysis, metaphors, addressing musical structure	4 Tutors 10 Students aged 7–15 (cello, trumpet, violin, and voice)	Modeling main strategy.
Broomhead, 2006	Case studies: interviews and lesson observation	Enquiry, student-initiated input, conducting, metaphors, modeling, feedback, verbal instruction	3 Conductors leading secondary school choirs	Instruction seemed instinctive and spontaneous.
Chester, 2008	Experimental study. Assessments evaluated articulation, dynamics, ritardando	Aural modeling, concrete verbal teaching, metaphors, no instruction	51 Students aged 10–13	No significant differences.
Dalaire, 2020	Intervention study. Assessments evaluated articulation, dynamics, ritardando	Dalcroze exercises	9 Pianists aged 5–12	No significant effect.
Ebie, 2004	Intervention study. Assessments evaluated intended emotion (happiness, sadness, anger, fear).	(1) Concrete verbal instruction, (2) aural modeling of a different melody, (3) kinesthetic exploration, (4) audio-visual imagery	56 Singers aged 12–15	Concrete verbal instruction less effective than other strategies. Note: all participants experienced all methods.
Griffiths, 2017	Auto-ethnography and case studies: interviews and video-recordings of lessons	Expressive gestures	4 Pianists aged 8–12	Expressive gestures not always useful.
Lisboa, 2000	Intervention study. Assessments evaluated overall expressiveness, performance (accuracy and fluency), and general understanding (of structure and phrasing).	No instruction, analysis, multi-modal approach (listening to expressive performance; singing before playing; coloring the score; discussing general musical issues)	3 Cellists aged 9–14	Multi-modal approach worked best. Note: all participants experienced all methods.
McPhee, 2011	Case studies: interviews and lesson observation	Metaphor, discussion of dynamics and articulation, phrasing, expressive markings in the score, own recordings	2 Tutors 4 Teenagers (cello and trumpet)	Various strategies useful. No significant differences.
Meissner, 2017	Exploratory action research study. Assessments evaluated overall expressiveness.	Aural modeling, enquiry and discussion, gestures and movements, imagery, “own” recordings, singing, “projected performance”	9 Tutors 14 Students aged 9–15 (flute, percussion, piano, recorder, violin, and voice)	Various strategies useful. No significant differences.
Meissner and Timmers, 2019	Experimental study. Assessments evaluated emotional expression, overall expressiveness, phrasing, accuracy, technical fluency, bodily communication.	Enquiry and discussion vs. teaching focused on improving accuracy and technical fluency	29 Students aged 8–15 (baritone, cello, clarinet, cornet, double bass, flute, French horn, piano, recorder, trumpet, and violin)	Enquiry and discussion significantly more effective for improving expressiveness in extract with “sad” character than control teaching.
Meissner et al., 2020	Video-stimulated recall interviews	Enquiry and discussion, improvisation, practice of difficult sections, scales practice	16 Students aged 8–15 (cello, clarinet, cornet, double bass, flute, piano, recorder, trumpet, and violin)	Enquiry and discussion perceived as useful. Practice of difficult sections and scales seen as useful too.
Meissner and Timmers, 2020	Participatory action research study	Aural modeling, enquiry and discussion, playing along	5 Tutors; 11 Students aged 8–15 (clarinet, cello, flugelhorn, French horn, piano, recorder, and violin)	Enquiry and discussion combined with aural modeling perceived as central for teaching and learning of EMP.
Vandewalker, 2014	Experimental study Assessment evaluated dynamics, tempo and note duration.	Aural modeling, metaphors, concrete verbal instruction	60 Wind band players aged 11–13.	Aural modeling and metaphors significantly more effective than concrete verbal instruction.

McPhee (2011) interviewed two tutors and analyzed video-recordings of brass and cello lessons with two teenagers and noted that teachers used various strategies: verbal metaphor; Discussion of dynamics and articulation; Consideration of phrasing; Expressive markings in the score; Own recordings; Giving students some choices regarding expressive characteristics of the music. McPhee observed that these strategies achieved similar outcomes providing students comprehended how their playing changed. Interestingly, she observed that students’ final performances followed teachers’ rather than learners’ interpretations. McPhee proposed that teachers could allow teenage students some responsibility for making interpretative decisions. Likewise Graham (1998) recommended that teachers and learners discuss the interpretation of a musical work to encourage students’ creativity in performance.

Broomhead (2006) observed and interviewed three teachers who were well-known for eliciting expressivity in secondary school choirs. He found that various instructional strategies were employed for facilitating expressiveness including enquiry; student-initiated input; metaphors; modeling; verbal feedback and detailed verbal instruction. Broomhead noted that teaching seemed instinctive and spontaneous and that a systematic approach to teaching expressiveness was missing. Broomhead proposed that *teachers’ enquiry*, providing problem-solving opportunities and encouraging students’ initiatives, can be used to facilitate teenagers’ expressivity in choir rehearsals (Broomhead, 2005). He observed that modeling and conducting consisting of expressive gestures combined with verbal explanations makes students dependent on their teacher-conductor. Therefore, he recommended that music educators use a constructivist approach for teaching performance expression and suggested that singers need opportunities to make expressive decisions to develop their understanding of EMP. According to Broomhead, the most productive environment for learning expressiveness is one where students both identify and solve problems of expression (2005).

In his book *Teaching Music Musically* Swanwick (2011) also recommends that there should be some scope for students’ choice, decision-making, and personal exploration in music education (p. 47). Based on the premise that music is symbolic discourse, Swanwick (2011) proposed three core principles for “teaching music musically”: care for music as a form of symbolic discourse^8^; attention for students’ contribution to musical participation; and the promotion of fluency, i.e., facilitating the ability to share, produce and cooperate in musical participation.

#### Intervention Studies Investigating Young Musicians’ Learning of Expressiveness

Some small-scale studies investigated the effectiveness of various instructional approaches on children’s performance expression during short experimental sessions. Chester (2008) compared aural modeling, concrete verbal instruction, verbal explanation using metaphors, and no instruction, on young musicians’ (aged 10–13) expressiveness in a one-off experiment but found no significant differences. Vandewalker (2014) conducted a short experimental study comparing aural modeling, verbal teaching using metaphors and concrete verbal instruction and found that aural modeling and verbal teaching using metaphors were significantly more effective than concrete verbal instruction for changing students’ (aged 11–13) use of dynamics, tempo and note duration. Both studies were based on short interventions and assessments of a few expressive cues. Neither overall expressiveness, nor expression of character was assessed leaving scope for further investigation.

Ebie (2004) compared the effectiveness of four teaching conditions on 56 teenagers’ (aged 12–15) emotional expression in singing: (1) “Concrete verbal instruction” explaining how to use their voices to sing expressively; (2) “Aural modeling” of a pre-recorded vocal track of a different melody with the intended emotional expression; (3) “Kinesthetic exploration” encouraging participants to explore musical emotions in a “physical and active way,” by acting, drawing, walking or moving around; (4) “Audio-visual learning” consisting of viewing 20 pictures while listening to a musical work that was expressive of the emotion that was conveyed in the pictures. Ebie found that the effectiveness of concrete verbal instruction was significantly lower than that of aural modeling and audio-visual learning, while the other three teaching conditions were similar in effectiveness for improving emotional expressiveness. Although Ebie’s aural modeling method seems less straight forward than conventional modeling where students hear a performance of the music they are learning, it had been effective for improving expressiveness. Perhaps the aural modeling, kinesthetic exploration and audio-visual learning conditions in this study were similar in effectiveness because they all referred to emotion, whereas the concrete verbal instruction focused on technical aims. However, in this study there could have been an effect of learning or boredom on expressiveness because each participant performed the test melody 16 times; with four different expressions (happiness, sadness, anger and fear) after four different teaching strategies.

Lisboa (2000) compared the effect of three instructional conditions on the practice and performance of three cello pupils (aged 9–14). She compared the following approaches: “no instruction,” “analytical instruction,” and a “multi-modal approach” which included singing before playing; coloring the score; watching a video of a professional cellist; and discussions of general musical issues, such as interpretation and approaches to practice and performance. Lisboa observed that her students concentrated on notation and fingering when left to their own devices (see also Pitts et al., 2000; McPherson and Renwick, 2001; Austin and Berg, 2006; Pike, 2017) and that the multi-modal approach led to the most expressive performances. It was not possible to determine whether the combination of complementary methods of the multi-modal approach had been especially effective, or whether one of these methods was successful, as four new strategies were introduced at the same time.

A different approach was taken in an experimental study with 155 teenagers (aged 12–15) by Broomhead et al. (2012), who explored the effect of confidence enhancing pre-performance routines (PPR) on singers’ expressiveness. The intervention for the experimental group consisted of repetition of “positive mindset trigger words” (bold, confident, and free) combined with breathing exercises and various small group performing activities, while control group participants attended normal choir rehearsals. Participants’ performances were assessed individually at baseline, post-intervention and after a 2-week period without intervention. The researchers found a significant difference between the experimental and control group for overall expressiveness scores immediately after the intervention but not after the last assessment. Furthermore, for the experimental group there was a significant difference between the first and the second assessment for overall expressiveness scores, but no significant difference was found between the first and the third test. These results suggest that a combination of confidence enhancing PPR, breathing exercises and regular performing activities can increase confidence and expressiveness in teenagers’ singing performance (Broomhead et al., 2012). Next, in an intervention study, classroom music teachers, after instruction from a psychologist, taught 132 teenagers confidence enhancing PPR for 3 weeks (Broomhead et al., 2018). The intervention provided students with performing opportunities containing “graduated levels of threat” (p. 62) to practice PPR. Results suggested that the intervention had a significant effect on students’ overall expressiveness immediately after the intervention, and also after 3 weeks without PPR instruction. In both studies *Happy Birthday* was used as test piece and the PPR were practiced in combination with breathing exercises and expanded performance activities. Each of the components of these interventions might have been effective or the combination of PPR and performance practice could have been useful. For future research, it would be worthwhile to explore the effects of confidence enhancing PPR and recurring performing activities on young musicians’ expressiveness in instrumental performance.

#### Movements and Gestures for Facilitating Young Musicians’ Learning of Expressiveness

Since interactions with music such as listening and performing are based on bodily activities that influence musical perception and meaning formation, several music pedagogues and scholars have emphasized the importance of an *embodied approach* through movements and gestures for children’s learning of EMP (e.g., Davidson et al., 2001; Juntunen and Hyvönen, 2004; Seitz, 2005; Leman, 2010, 2017; Lesaffre et al., 2017; Davidson, 2018; Nijs, 2018; Schiavio et al., 2019). The concept of “embodiment” refers to “human interaction as expressed through corporeal articulations and body movements” (Leman et al., 2017, p. 1). An example of an embodied approach “avant la letter” can be found in instructional strategies based on Jacques-Dalcroze’s ideas. In Dalcrozian instructional strategies kinetic exercises link musical parameters such as tempi, durations, pitches, rhythms and tonalities with physical movement, to provide students with their own working knowledge of these concepts (Johnson, 1993).

The article by Davidson et al. (2001) contains an interesting vignette taken from a lesson with a 9-year-old pianist to illustrate the use of an embodied approach. This vignette demonstrates how movements and gestures can be combined with dialogic teaching consisting of open questions and dialogue. First the teacher asked an open question about the musical character: “What do you think about this piece?” Afterward, in a dialogue between teacher and pupil, the girl suggested that the piece is about “a Queen or a Princess who is all Royal but sad and then happy.” The teacher recommended that she moves around as if she were the princess to explore the music. In this example open questions, dialogue and reflection were combined with movements and gestures to explore the musical character (Davidson et al., 2001, p. 20).

Griffiths (2017) conducted year-long case studies with four of his piano students (aged 8–12) exploring how expressive gestures might be used for developing expressive performance skills. He concluded that expressive gestures are not always an appropriate teaching strategy for every learner as instruction should be adjusted to each individual student, because of differing motor skills, kinesthetic awareness, technique and practice habits, and ought to build up confidence first.

Fortuna and Nijs (2020a, b) compared the effects of movement-based activities (“show the music in body movements”) with verbal-based activities (“describe the music verbally”) on children’s (aged 9–10) graphical depictions of a composition (“describe the music with pencils”). After listening to *Kangaroos* by Saint-Saëns and drawing a representation of the music, the children were asked to explain their drawings in words. The pre- and post-intervention drawings were analyzed for differentiated and global representations, with differentiated representations depicting one or more musical parameters and global representations portraying metaphorical or narrative interpretations of the music. The results of this study showed a significant increase of differentiated representations from pre-test to post-test in drawings of children involved in listening combined with movement-based activities. Participants involved in movement-based activities used more abstract shapes, lines and analogous images in their post-test drawings than participants involved in verbal-based activities. Furthermore, the analysis indicated that children involved in listening combined with verbal-based activities used more varied elements in their post-test global drawings, such as symbols, shapes, and figurative images (e.g., castles and wolves), than at the start of the intervention. These findings suggest that learning activities based on different modalities of interaction affect the development of musical meaning making in different ways (Fortuna and Nijs, 2020a,b). It is important to note that the verbal learning activities in this study were not dialogic teaching strategies. In the study by Fortuna and Nijs children were invited to “find different words and their opposites” to describe music, to describe the music in writing, to explain the rationale behind their classmates’ written descriptions, and afterward, they were encouraged to share their interpretations with classmates. In contrast, in dialogic music teaching open questions and spoken dialogue are employed to stimulate thinking and to develop understanding and meaning making (see section Dialogic Teaching Approach for Developing Children’s Expressiveness”). The findings of this study are of interest for teaching and learning of expressiveness, as these show that various modalities can affect children’s meaning making in music in different ways.

Dalaire (2020) investigated the effects of Dalcroze training on Suzuki piano pupils’ (aged 5–12) expressivity by measuring variations in dynamics, timing and articulation collected via MIDI data. Participants played a piece they had learned previously in two pre-tests and two post-tests. The intervention consisted of 3 h-long Dalcroze sessions on three consecutive days by a certified Dalcroze teacher. Data analysis did not show a significant effect for the Dalcroze exercises on young pianists’ use of dynamics, timing, or articulation. This was a small-scale project and it would be worthwhile to investigate the effect of Dalcroze training further in long-term studies with larger samples, perhaps including children who are not Suzuki trained as the aural model used during training might have influenced the outcome of the study.

The use of movements and gestures for facilitating expressiveness was also investigated by a teacher in my exploratory action research (AR) project (Meissner, 2017). In this project nine instrumental teachers, including the author, explored various methods for facilitating young musicians’ (aged 9–15) expressiveness in performance. The tutors in this study found that a range of methods can be employed for developing children’s expressiveness including enquiry and discussion, movements and gestures, modeling, visual imagery, thinking of drama, listening to “own” performances, and “projected performance.” Tutors in this project emphasized that young musicians should learn to “think for themselves” and “own the performance.” One of the flute teachers used bodily movements in various ways: With one pupil she used arm movements to explore phrasing and dance movements to express the musical character. With another learner she used gestures as feedback; while the student was playing the teacher made arm movements to mirror the character conveyed by the student’s playing. The gestures and movements used by this tutor are similar to bodily movements recommended by Davidson (2018) for teaching EMP. The flute teacher observed that, although the gestures exploring phrasing and dance movements expressing character had generated an expressive performance during the lesson, this effect had disappeared in subsequent lessons. Furthermore, the participating teachers thought that all the instructional strategies used had been helpful in lessons. However, assessments of performances did not show a significant development in students’ expressiveness, although results suggested that enquiry and discussion may be effective for improving young musicians’ expressiveness (Meissner, 2017). As this was an exploratory study with limited participants more research was required into the use of questions and enquiry for teaching and learning of expressiveness (see below).

#### Summary

In summary, research has found that children tend to focus on technique and reading from notation during practice (e.g., Lisboa, 2000, 2008; Pitts et al., 2000; Pike, 2017). Several small-scale studies observed instructional strategies for teaching performance expression (Broomhead, 2006; McPhee, 2011; Brenner and Strand, 2013) or explored the effectiveness of various teaching methods (e.g., Lisboa, 2000; Chester, 2008; Vandewalker, 2014; Meissner, 2017). Overall, these studies found that various instructional strategies can be used to enhance children’s expressiveness such as aural modeling, enquiry and discussion, metaphors and visual imagery, movements and gestures, listening to “own” performances, and “projected performance.” Additionally, it may be beneficial for students to construct their understanding of EMP via problem solving activities (Broomhead, 2005; Meissner, 2017). Generally, these were projects with small groups of participants that have not established a reason yet for the limited expressiveness of some young musicians’ performances, leaving scope for further research. Scholars advocating the use of movements and gestures for developing expressivity (e.g., Davidson et al., 2001; Juntunen and Hyvönen, 2004; Davidson, 2018; Nijs, 2018) have suggested a rationale for using bodily movements in music education but more research is required to explore the effectiveness of gestures and movements for facilitating children’s learning of EMP.

### Dialogic Teaching Approach for Developing Children’s Expressiveness

It was my aim to explore instructional strategies for facilitating young musicians’ expressiveness further to contribute to a systematic pedagogy for teaching and learning of EMP. As findings from my exploratory AR study suggested that enquiry and discussion of musical character and expressive devices may be helpful for improving young musicians’ expressivity (Meissner, 2017) this required further investigation. Enquiry and discussion are central aspects of a dialogic pedagogy approach. Dialogic teaching relates to instruction that is characterized by open questions and dialogue rather than teacher presentation (Alexander, 2008, 2010). Thus, dialogic teaching of expressive performance is characterized by teacher enquiry and discussion to stimulate and extend learners’ thinking about the musical character and structure, and how to convey this in performance (Meissner, 2018; Meissner and Timmers, 2019). Three studies were organized to explore the use of dialogic teaching for facilitating young musicians’ learning of EMP: an experimental vs. control group study in conjunction with a qualitative study consisting of a questionnaire and video-stimulated recall interviews (VSRI) and a participatory action research project with four colleagues.

In the experimental study 29 young musicians (20 girls and 9 boys, aged 8–15) took part in an improvisation test followed by an experimental vs. control group lesson. The aim of the improvisation test was to explore whether participants had knowledge about the use of expressive cues, such as articulation, dynamics, and tempo, for conveying basic emotions at the start of the study. Adjudicators’ assessments indicated that most participants could convey happiness, sadness, and anger effectively in improvisations. Improvisations conveying sadness were most successful (90%), followed by improvisations portraying happiness (79%), whilst anger was more challenging to convey or recognize successfully. The average scores for the use of musical content (MC) were significantly higher than those for the use of expressive cues (EC). EC scores were not high, implying that these participants had some, possibly intuitive, knowledge concerning the purpose and application of expressive tools, which can be a useful starting point for teaching-and-learning expressivity. Age and level of playing did not correlate with participants’ use of EC (Meissner, 2018; Meissner and Timmers, 2019).

The experimental study investigated whether questions and discussion regarding the musical character and the use of expressive devices is more effective for improving learners’ expressiveness than instruction focusing on accuracy and technique. Video-recordings of participants’ performances of two extracts portraying contrasting emotions (happiness and sadness) pre- and post-teaching were assessed by four adjudicators. Results indicated that the experimental teaching had been significantly more effective for improving emotional expression and overall expressiveness scores in the “sad” extract than control teaching, and there was a tendency for control teaching to be more effective for improving technical fluency and accuracy scores in the “happy” piece (Meissner and Timmers, 2019). Age and level of playing did not correlate with difference scores for emotional expression or overall expressiveness (Meissner, 2018).

In the second study all 29 participants filled in questionnaires immediately after the research session and 16 participants (11 girls and 5 boys) took part in VSRIs approximately a year later. The VSRIs explored young musicians’ perspectives on the instruction that was used during the experimental study, which included practice of difficult sections, scales practice, improvisation, and questions and dialogue regarding musical character. Most participants found the instructional strategies that had been used helpful and young musicians who had been taught via dialogic teaching reported that the questions relating to musical character and expressive devices had been useful for their understanding of the “musicality” of their pieces and thus for their learning of performance expression. The questions regarding musical character had facilitated their reflection on, and understanding of the interpretation, thus contributing to their learning of expressiveness. Findings from the experimental and the VSRI study demonstrate the usefulness of teachers’ enquiry and learners’ reflection for young musicians’ learning of EMP (Meissner, 2018, 2020; Meissner et al., 2020).

Subsequently, in a third study five instrumental music teachers, including the author, investigated teaching and learning of expressiveness in a participatory AR project with 11 girls (aged 8–15) playing various instruments. This project aimed (1) to explore how dialogic teaching and learning of expressiveness can be used in weekly instrumental lessons; (2) to investigate whether instrumental tutors find a dialogic teaching approach useful for facilitating young musicians’ learning of expressiveness; and (3) to explore which complementing instructional modes tutors would like to employ. Furthermore, this study aimed (4) to investigate young musicians’ views on their learning of expressiveness; and (5) their views on instructional strategies used for teaching expressiveness. Teachers in this AR project observed that instruction should be tailored to the individual student and situation. They pointed out that teaching and learning EMP is a complex process wherein “everything is intertwined”: instructional strategies that facilitate learning of expressiveness may also serve to develop accuracy or technical skills, while improved accuracy and technique in turn can enhance the expressiveness of a performance. The teachers explored mainly dialogic teaching, modeling, and playing along with students. Four teachers reported that aural modeling combined with dialogic teaching had been especially helpful, and some were impressed by the effectiveness of asking questions for facilitating expressiveness. Young musicians in this study reported that they had learned to think about the musical character and how to convey this in performance. Their teachers’ questions had stimulated reflection and raised their awareness of the musical meaning, while teachers’ modeling had helped to build up an aural picture of the music which had facilitated their learning. The dialogic teaching approach combined with modeling had generated enhanced expressiveness in lessons and contributed to a growing sense of achievement, confidence, self-efficacy, and musical agency. Although participants thought that pupils’ expressiveness had improved in lessons, this was not noticeable yet in performances, probably due to performance anxiety and the difficulty level of pieces. Nevertheless, participating teachers and students thought that the repeated performance experiences had helped to develop confidence (Meissner and Timmers, 2020).

To understand more about young musicians’ learning of expressive performance, pupils were given questionnaires to explore their views on the “hardest” and “best” features of learning to play a musical instrument and their approach to practice. Firstly, students’ responses indicated that learning to play a musical instrument was perceived as a challenging activity from the beginning of learning and that young musicians became more aware of the complexities of playing when they got older and started working on harder repertoire. If note reading and physical aspects of playing are difficult, this could be an obstacle for playing expressively (e.g., Lisboa, 2000; Broomhead, 2001). Secondly, participants in these studies reported that they tended to concentrate on reading from notation, “technicalities” and playing through pieces during practice, overlooking matters of interpretation because of the challenges of instrumental playing. This is in line with research that found that young musicians tend to have inefficient practice strategies and are inclined to play through pieces (e.g., Pitts and Davidson, 2000; Pitts et al., 2000; McPherson and Renwick, 2001, 2010; Austin and Berg, 2006; Lisboa, 2008; Hallam et al., 2012; Pike, 2017). Thirdly, questionnaire answers suggested that the challenges of instrumental music learning were balanced by the rewards of music participation. Among these participants there was an awareness of growing musical competence, a sense of achievement when progress was made, or goals reached. Additionally, some participants reported that they enjoyed the aesthetic aspect of music making; music itself was motivating these learners to continue their instrumental music learning (Meissner, 2018, 2020; cf., Pitts, 2005; Silverman, 2020).

To summarize, young musicians in these studies saw instrumental music learning as a challenging activity and they tended to focus on music reading, “technicalities” and playing through pieces in their practice prior to their research participation. Participants reported that various instructional strategies used during the experimental study, such as practice of difficult sections, scales practice, and questions and dialogue regarding musical character, had been useful. Furthermore, participants from these studies reported that listening to aural models and talking about the music had been helpful for their learning. According to these students, questions and discussion had facilitated their reflection on the “musicality” of their pieces and how to convey this, whereas before they had concentrated on the “technicality” of instrumental playing and viewed their music as “just notes” (Meissner, 2018; Meissner and Timmers, 2020). Participating teachers reported that questions and discussion combined with aural modeling had been useful for facilitating various aspects of students’ music performance, including expressiveness. Neither the assessment scores for expressiveness, nor the themes that emerged from questionnaire responses were correlated to participants’ age or level of playing.

## Theoretical Framework and “Toolkit” for Teaching Young Musicians Expressive Performance

Based on this body of research I propose a new theoretical framework for facilitating young musicians’ learning of expressive music performance. Firstly, if we define musicality as the ability to perceive, appreciate and produce music, we can assume that every child is musical (cf., Honing, 2009; Hoeschele et al., 2015; McPherson and Hallam, 2016). Since infants have innate gifts for musical communication (Honing, 2012, 2018; Tan et al., 2014; Háden et al., 2015; McPherson and Hallam, 2016) and experience with infant-directed speech and song (e.g., Trehub and Nakata, 2001; Trevarthen, 2002; Trehub, 2003), and because young children display expressive vocalizations and enjoy exploring musical sounds (Moorhead and Pond, 1942; Moog, 1976; Trehub and Nakata, 2001; Tafuri, 2008; Addessi, 2009; Delalande and Cornara, 2010), we can expect young musicians to have a repertoire of “expressive gestures” when they start formal instrumental music lessons (cf., Sloboda and Davidson, 1996; Trehub, 2003; Sloboda, 2005; Juslin and Timmers, 2010). This experience with musical play and communication from early childhood forms the foundation for EMP.

Secondly, it is important to understand *why* children tend to focus on technique and note reading during practice rather than on expression and communication (see Pitts and Davidson, 2000; Pitts et al., 2000; McPherson and Renwick, 2001; Austin and Berg, 2006; Lisboa, 2008; McPherson and Renwick, 2010; Pike, 2017), and why there seems to be a focus on these elements of instrumental playing in lessons too (see Rostvall and West, 2003, 2005; West and Rostvall, 2003; Karlsson and Juslin, 2008). The questionnaire findings reported above (Meissner, 2018) support the view that this focus on technique and notation in instrumental practice and lessons is generated by the difficulties which children experience when they start learning their instruments. Learning to play a musical instrument is a demanding process, as various skills need to be learned simultaneously (see e.g., Dowling, 1973; Chaffin et al., 2003; Hallam, 2006) while playing fluently is a long-term process requiring many hours of deliberate practice (e.g., Sosniak, 1990; Ericsson et al., 1993; Sloboda, 1996; McPherson et al., 2012). The instructional strategies used in the experimental study, such as practicing scales and difficult sections as well as enquiry and discussion had been helpful for participants because of the difficulties of playing an instrument. When handling an instrument is challenging and note reading seems complicated young musicians probably focus on technique and notation. This could easily lead to a vicious circle of students focusing on notes and technique, and teachers perceiving their students as “wooden” performers who need to improve accuracy and technical fluency first. Consequently, the challenges of instrumental playing may generate a focus on “technicality” and a lack of reflection on musical communication causing limited expressiveness in young musicians’ performance (Meissner, 2018).

**FIGURE 1:**
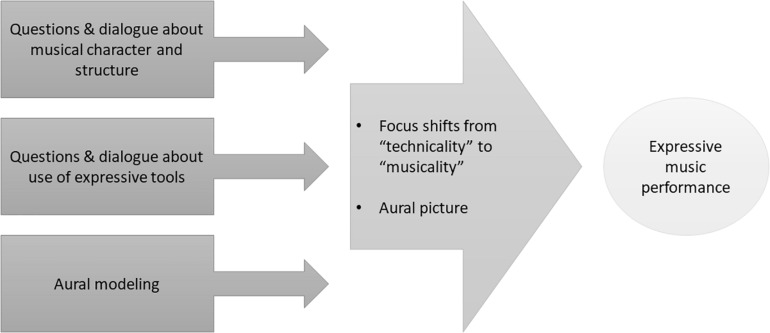
Theoretical model illustrating how open questions and dialogue combined with aural modeling can generate a focus on “musicality” thus increasing expressiveness in performance.

Thirdly, we need to understand *how* instructional strategies can be effective for facilitating children’s expressiveness in music performance. When children focus on technical difficulties or note reading rather than the expressive content of music, open questions can raise awareness of, and stimulate thinking about, the interpretation of the musical character and structure. Questions can help children to make a connection between the music they are playing and the emotions, movement, or stories that they may perceive in the music. By asking open questions about the musical character and the use of expressive cues, teachers can shift young musicians’ attention from a focus on the “technicality” to an increased awareness of the “musicality” of their pieces (Meissner et al., 2020) which may enhance expressiveness. Furthermore, teacher modeling or listening to a recording can provide an “aural picture” of the music which can be helpful for developing several aspects of music performance, including accuracy, technical fluency, phrasing and overall expressiveness (Meissner and Timmers, 2020). The interpretation heard in an aural model can be discussed and explored via questions and dialogue and by playing a composition in different ways (Figure 1). In addition, research has shown that various instructional strategies may be useful for developing children’s expressivity, such as movements and gestures, singing, and concentrating on “projected performance” (e.g., McPhee, 2011; Meissner, 2017). These strategies can support the development of various aspects of performance, such as phrasing, technique, and accuracy thus contributing to the overall expressiveness.

**FIGURE 2:**
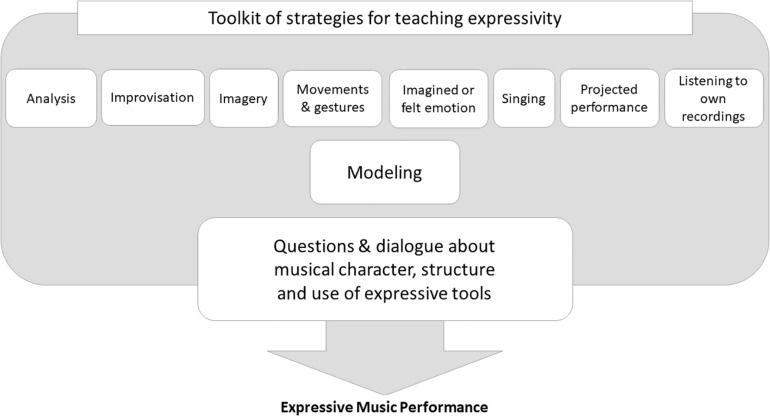
“Toolkit” of strategies for teaching expressive music performance.

Based on this theoretical framework and the research reported above I developed a “toolkit” of instructional strategies for teaching expressivity (Figure 2). This “toolkit” (cf., Ward, 2007) illustrates how a range of methods can complement a dialogic teaching approach. Open questions and discussion are central and serve to stimulate thinking, raise awareness of, and connect the musical ideas to the embodied experience of the learner. Modeling can be used to support this instruction and to provide an aural picture while various supplemental methods such as imagined emotion, movements, gestures, and “projected performance” can be used to illustrate and explore the musical character and structure further. For instance, children can create dance movements or arm gestures to explore and convey the character or direction of the music, or they can be invited to project their interpretation of the composition to an audience in an imaginary space.

**FIGURE 3:**
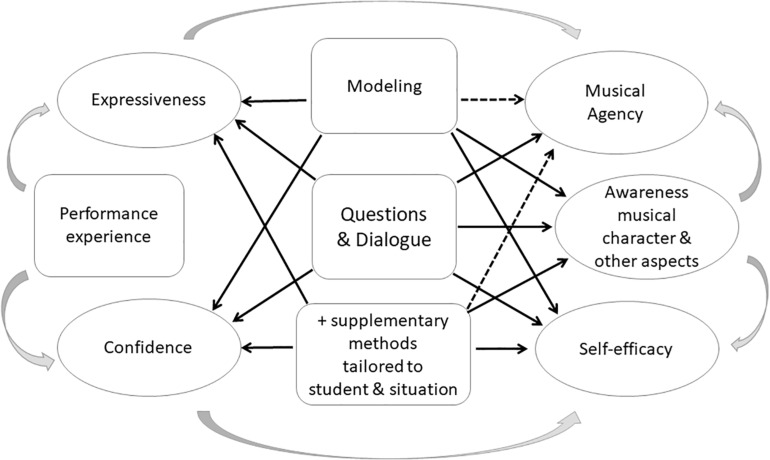
Theoretical model illustrating how “everything is intertwined” in teaching and learning of expressive music performance.

Fourthly, teaching-and-learning of expressive music performance is a multifaceted interactive process wherein “everything is intertwined” (Figure 3). Effective dialogic teaching combined with modeling can facilitate the development of young musicians’ expressiveness, confidence, self-efficacy and musical agency (Meissner and Timmers, 2020). Performance experience is significant for the learning process too, as research has demonstrated that performing regularly can increase students’ confidence (Meissner and Timmers, 2020; cf., Broomhead et al., 2012, 2018) and that performing needs to be trained (e.g., Kenny and Ackermann, 2016; Papageorgi and Kopiez, 2018; Yandell, 2018). Developing expressiveness, performance experience and confidence take time and practice. In addition, many other aspects, including the instrument played, level of difficulty and style of the music (Meissner, 2018), personal factors such as motivation and confidence of student and teacher, music preferences and listening experiences, potential learning difficulties (cf., Ockelford, 2000; Overy et al., 2003), creativity, personality and interactional style of teachers and students (Creech, 2012) are likely to affect the process of teaching-and-learning EMP. Although it is important to understand the separate components of the teaching and learning process, it is not sufficient when we understand one part of this complicated process. We should be aware of the complexity of children’s musical development as it is embedded in teachers’ and students’ experiences and environment (cf., Silverman, 2020). Therefore, instruction should be tailored to the individual learner and situation.

## Discussion and Conclusion

This article set out to propose a new theoretical framework for teaching and learning of expressive music performance based on a comprehensive review of the literature related to this topic. The presented framework provides a plausible reason for the occurrence of limited expressiveness in some children’s performances, i.e., the challenges of instrumental music learning inducing a focus on notation and “technicality,” and the framework also proposes ways to facilitate learners’ expressiveness via a dialogic teaching approach combined with aural modeling. Recent studies have revealed that dialogic teaching consisting of enquiry and discussion is effective for enhancing children’s expressiveness as this can raise students’ awareness of and stimulate thinking about the musical character and how to convey this in performance. Young musicians reported that their teachers’ questions regarding the musical character were “eye-openers” as these served to shift their focus from the “technicality” to the “musicality” of pieces (Meissner and Timmers, 2019; Meissner et al., 2020).

Combining dialogic teaching with aural modeling was considered central by teachers and students for developing expressiveness (Meissner and Timmers, 2020) as aural models can provide learners with an aural picture of the music (Rosenthal, 1984; Sang, 1987; Dickey, 1992; Woody, 2006a), thus facilitating their learning of all aspects of EMP, including accuracy and technical fluency, phrasing and expressiveness. Teachers and students can listen to performances together and discuss the interpretation and expressive tools that might be used. Aural modeling can support and illustrate the teacher-student dialogue about the music. It is important to emphasize that modeling without enquiry and discussion is less useful than aural modeling combined with dialogic teaching, as modeling alone might make students dependent on their teacher, while questions can stimulate students’ thinking about the interpretation heard in an aural model (Meissner and Timmers, 2020). This is in line with findings by Woody (2003) that pianists who could describe what they had heard in an aural model were more likely to copy the model correctly than pianists who were unable to give a verbal description. Reflecting on and discussing an aural model can support the construction of one’s own interpretation about a composition.

Additionally, methods such as gestures and movements, projected performance, playing along with pupils, or singing may be used within a dialogic teaching approach. Although such supplementary instructional strategies can be useful, their effectiveness might be limited if used without questions that stimulate thinking about, and raise awareness of, the musical character and structure. Movements and gestures for example might be more effective when they are combined with dialogic teaching and modeling than without this. Although children might feel the music’s direction and character in their bodies while moving this does not necessarily imply that they can translate these feelings into expressive devices for music performance (cf., Dalaire, 2020). However, combining movements and gestures with questions and dialogue is likely to raise awareness and stimulate thinking and learning. Future research may explore the use of movements and gestures within a dialogic teaching approach further.

Tutors’ perceptions of musical meaning and interpretation are influential for their approach to teaching expressiveness. For dialogic teaching of EMP, it is crucial to realize that musical meaning comes into existence when it is performed and heard and tends to vary depending on the performer and situation. This characteristic of musical meaning offers tutors the opportunity to ask authentic open questions about the interpretation and to invite students to reflect on their personal interpretation of the meaning of a composition. According to McPherson et al. (2012), the lack of communicative and expressive content in lessons, and failure to transform students’ musical participation into a meaningful and personal activity were causes for the high drop-out rate observed in their sample. Via open questions about the musical meaning teachers can provide learners with opportunities to connect the music they are learning to their own personal experience.

Asking questions about the musical interpretation may seem an obvious approach for teaching expressivity. However, reactions from participants demonstrated that the questions used in the experimental study and the AR lessons were new for these students and revealed to them the possibilities of thinking about and deciding on the interpretation of the musical character (Meissner, 2018). Additionally, some tutors were impressed by the effectiveness of asking questions for facilitating expressivity, suggesting that this was a new approach for them too (Meissner and Timmers, 2020). Moreover, researchers who observed teaching and learning of expressiveness in instrumental lessons (West and Rostvall, 2003; Karlsson and Juslin, 2008; McPhee, 2011; Brenner and Strand, 2013) did not mention that questions were used for stimulating thinking or facilitating expressivity. Overall, this suggests that open questions about the interpretation and how to convey this, might not be common practice in instrumental music tuition for facilitating young musicians’ expressivity.

Questions and dialogue are important instructional tools for a constructivist approach to teaching and learning. In the constructivist theory the teacher is seen as the facilitator of learning; teachers design learning activities in which learners construct their own understanding by combining and reorganizing pre-existing knowledge (e.g., Broomhead, 2005; Kauchak and Eggen, 2012; Von Glasersfeld, 2012). Several scholars have emphasized that questions and dialogue can be important tools for the development of understanding (e.g., Mercer, 1995; Alexander, 2008; Kleine Staarman and Mercer, 2010). According to Vygotsky language is crucial for the development of concepts and thought: “Thought is not merely expressed in words; it comes into existence through them. Every thought tends to connect with something else, to establish a relation between things. Every thought moves, grows and develops, fulfills a function, solves a problem” (Vygotsky, 1986, p. 218). According to Vygotsky understanding is constructed through people’s spoken language and their involvement in social events (Vygotsky, 1978, 1986). A problem-posing approach and social interaction are important elements of dialogic pedagogy too, as open questions invite students “to participate actively in reshaping their own understanding of reality” (Skidmore and Murakami, 2016, p. 13, referring to Freire, 1993). According to Alexander (2010) dialogic teaching “harnesses the power of talk to stimulate and extend pupils’ thinking and advance their learning and understanding. It helps the teacher more precisely diagnose pupils’ needs, frame their learning tasks and assess their progress” (p. 1).

It is important to note that dialogic teaching is not the same as “mere” verbal instruction which could be a barrier for music learning (see Juntunen and Hyvönen, 2004; Bannan, 2005). Concrete verbal teaching that is detached from children’s experience, or verbal instruction prescribing an interpretation might well be unhelpful for children’s musical development. However, dialogic teaching that uses authentic open questions is fundamentally different to verbal teaching, as questions can help learners to make a connection between the music and their embodied experiences. Accordingly, dialogic teaching can be employed in a “4E” approach to music education.

In a 4E account, cognitive processes are perceived as *embodied, embedded, enacted*, and *extended* (e.g., Schiavio et al., 2017; Schiavio and van der Schyff, 2018; Schiavio, 2019; Silverman, 2020). Dialogic teaching of EMP provides a way for learners to connect the musical character to their own *embodied* experiences of the music they are playing. Asking open questions can raise the awareness of the musical meaning and involves the whole person in learning. Open questions and dialogue employing metaphors can help children to make a connection between the music they are learning and action-states, feelings, narratives, or characters that may be expressed in the music (cf. Abrahamson, 2020). Since music has the potential to evoke affect, character, or emotion, we can talk metaphorically about the music. In addition, we tend to talk metaphorically about affects and emotions; “All feeling states have their own patterns of activity, their own mix of weight, space, time and motion” (Swanwick, 2011, p. 17). Young musicians’ ideas about the musical character are *embedded* into the contexts of their own musical experiences and surrounding worlds (cf., Silverman, 2020). Via dialogue and exploration young musicians can develop their ideas and understanding of the musical works they are learning, improvising, or composing, thus *enacting* their identity as musicians. Furthermore, young musicians can *extend* their music making in the interaction with others in ensemble work, via dialogue in group lessons and via improvisations and compositions.

When instrumental music learning is challenging children tend to focus on the “technicality” of their playing. For effective *scaffolding^9^* of the learning process, teachers should have a thorough knowledge of their student and their level of task mastery (Wood et al., 1976). This implies that instrumental music teachers should be aware of the difficulties of instrumental playing and the challenges of pieces: they need to understand whether a composition is of an appropriate level for their student at this point in their learning process. It is important to find pieces that are exactly the right level of difficulty as young musicians appreciate having a challenge (Custodero, 2002), but working on pieces that are too difficult can be frustrating and may hinder work on expressiveness, while pieces that are too easy might not be inspiring either (see Meissner, 2018). Having the right balance between challenge and current task mastery is essential for the scaffolding process of teaching-and-learning expressiveness (Wood et al., 1976).

Research findings suggest that it is possible to work on EMP at all stages of learning and all ages. Even when children are in the early stages of learning a piece it is possible to work on expressiveness via basic questions about the musical character as this helps the learner to place the piece into a meaningful context (cf., Meissner et al., 2019; Meissner and Timmers, 2020). Beginners can be encouraged to consider the musical character of their pieces and they can be invited to improvise tunes conveying basic emotions or sound effects. This will help them to connect their music learning to their embodied experiences and will serve to make their musical participation personal and meaningful. From the early stages of learning teachers can provide young musicians with the opportunity to make their own interpretative choices, thus supporting their musical agency (see Wiggins, 2016).

Some scholars suggested that children are fully occupied with handling their instrument in the early stages of learning and that learners should first learn the basics required for playing a piece before working on EMP due to the complexity of the tasks involved (cf., Lehmann et al., 2007; McPherson et al., 2012; Davidson, 2018). On one hand this is a sensible approach, as research indicates that technical difficulties can hinder expressiveness (Broomhead, 2001; Meissner, 2017; Meissner and Timmers, 2020) and the cognitive load theory suggests that all learning tasks have an inherent difficulty or cognitive load (Sweller, 1988). Various aspects of instrumental performance, including music reading, motor control and matters of interpretation, may contribute to cognitive load (cf., Owens and Sweller, 2008; Stambaugh, 2013). However, on the other hand, some studies suggest that it is possible to work on expressive performance at every level of learning and that focusing on the musical character may even benefit young musicians’ accuracy and technical fluency in some instances (Meissner and Timmers, 2019, 2020; Meissner et al., 2019). This is in line with research that demonstrates that an external focus of attention, directed at movement effect, enhances motor performance and learning (Wulf et al., 2010, 2016; Wulf, 2013). Therefore, an external focus on expressiveness and communication may benefit young musicians’ accuracy and technical fluency. Music teaching stimulating an external focus during performance is likely to speed up the learning process (Wulf, 2013; Mornell and Wulf, 2019).

Teaching-and-learning of expressive music performance is a multifaceted interactive process wherein “everything is intertwined”: instructional strategies and teaching aims; level of difficulty and style of the music; motivation and confidence of student and teacher; music preferences and experiences of student and teacher; instruments played; performance practice and experience; pre-performance routines (Broomhead et al., 2012, 2018; Hawkes, 2018); and personality and interactional style of teachers and students (Creech, 2012). It is important to be aware of these factors and to adjust instruction and music to the student and situation. More empirical research is required to explore the feasibility of the proposed theoretic framework for teaching and learning of EMP.

Future research could also investigate teaching and learning of expressive intensity and musical tension in young musicians’ performance. It might be possible to raise teenagers’ awareness of musical tension, and how to create expressive intensity, via questions and discussion supported by aural modeling or gestures, or to discuss the expressive intensity heard in recordings. It would be worthwhile exploring from which age or level of playing it is possible to work on musical tension, and whether teenagers can explore this aspect of performance in their ensemble and improvisation work too.

In conclusion, the presented dialogic teaching framework for facilitating young musicians’ performance expression provides tutors with practical instructional tools as well as a rationale for using these. Scaffolded dialogic teaching consisting of open questions and dialogue combined with aural modeling can stimulate young musicians’ thinking, enhance their expressiveness, and contribute to their sense of achievement, autonomy, self-efficacy and musical agency. Such transformative teaching facilitates students’ creative problem solving skills and fosters independent thinking (cf. Broomhead, 2005; Creech and Gaunt, 2018). If learners additionally have frequent performing opportunities, this combined approach is likely to provide tutors with a rewarding teaching practice and young musicians with training that enables them to flourish; to play expressively and find their own “musical voice.”

## Data Availability Statement

The original contributions generated for this study are included in the article/supplementary material, further inquiries can be directed to the corresponding author.

## Ethics Statement

No new data involving human subjects was gathered toward writing this paper. Where previously published empirical work is cited that was conducted by HM all ethical standards were met therein in accordance with the requirements of the Ethics reviewers at The University of Sheffield.

## Author Contributions

The author confirms being the sole contributor of this work and has approved it for publication.

## Conflict of Interest

The author declares that the research was conducted in the absence of any commercial or financial relationships that could be construed as a potential conflict of interest.

## Footnotes

1 *Expressiveness* refers to the expressive intensity and quality of a music performance (Meissner, 2018; Meissner and Timmers, 2020; Meissner et al., 2020).

2 Whenever *instrumental music teachers* are mentioned voice teachers are included.

3 In this article ‘*we*’ is intended to refer to people in general, particularly the readers who might be interested in the topic of this paper.

4 In this article *emotion* is used in a generic sense, including affect, feeling and mood.

5 Tagg’s definition is accompanied by eight axioms and three tenets.

6 Here *musical meaning* refers to the composer’s intentions and/or the performer’s interpretation of the musical character and structure of a work. Musical meaning making is related to the 4E (enacted, embodied, embedded, and extended) concept of *sense-making* as described in Silverman (2020).

7 *Teaching methods* or *instructional strategies* relate to tutors’ actions that aim to promote and facilitate student learning.

8 Based on work by philosophers such as Cassirer (1955), Langer (1957), and Swanwick (2011) proposed that “the dynamic phenomenon of metaphor” underlies musical discourse. He suggested that musical metaphor contains *likeness*, or a *resemblance* rather than a reproduction of feelings, motion, and tension (p. 13).

9 *Scaffolding* describes the interactive process of tutoring in which the teacher controls those elements of the task that are initially beyond the learner’s capacity, thus permitting the learner to concentrate upon and complete only those elements that are within their range of competence (Wood et al., 1976, p. 90).

## References

[B1] Abrahamson D. (2020). Strawberry feel forever: understanding metaphor as sensorimotor dynamics. *Senses Soc.* 2 216–238. 10.1080/17458927.2020.1764742

[B2] Addessi A. R. (2009). The musical dimension of daily routines with under-four children during diaper change, Bedtime and free-play. *Early Child Dev. Care* 179 747–768. 10.1080/03004430902944122

[B3] Alexander R. (2008). *Towards Dialogic Teaching: Rethinking Classroom Talk*, 4th Edn Osgoodby: Dialogos UK Ltd.

[B4] Alexander R. (2010). *Dialogic Teaching Essentials.* Available online at: http://www.nie.edu.sg/files/oer/FINAL Dialogic Teaching Essentials.pdf (accessed December 16, 2020).

[B5] Ashley R. (2017). “Music and communication,” in *The Routledge Companion to Music Cognition*, eds Ashley R. Timmers R. (New York, NY: Taylor & Francis Group), 479–488. 10.4324/9781315194738-39

[B6] Austin J. R. Berg M. H. (2006). Exploring music practice among sixth-grade band and orchestra students. *Psychol. Music* 34 535–558. 10.1177/0305735606067170

[B7] Bannan N. (2005). *Music Teaching Without Words. Electronic Musicological Review*, *IX.* Available online at: https://www.researchgate.net/publication/239602378 (accessed December 16, 2020).

[B8] Barten S. S. (1992). The language of musical instruction. *J. Aesthet. Educ.* 26 53–61. 10.2307/3332923

[B9] Bonastre C. Muñoz E. Timmers R. (2017). Conceptions about teaching and learning of expressivity in music among Higher Education teachers and students. *Br. J. Music Educ.* 34 277–290. 10.1017/S0265051716000462

[B10] Bonastre C. Timmers R. (2019). Comparison of beliefs about teaching and learning of emotional expression in music performance between Spanish and English HE students of music. *Psychol. Music* 8:030573561984236 10.1177/0305735619842366

[B11] Brendel A. (2011). *On Character in Music.* Available online at: https://www.medici.tv/en/masterclasses/alfred-brendel-on-music-episode-2-musical-characters/ (accessed December 16, 2020).

[B12] Brenner B. Strand K. (2013). A case study of teaching musical expression to young performers. *J. Res. Music Educ.* 61 80–96. 10.1177/0022429412474826

[B13] Broomhead P. (2001). Individual expressive performance: Its relationship to ensemble achievement, technical achievement, and musical background. *J. Res. Music Educ.* 49:71 10.2307/3345811

[B14] Broomhead P. (2005). Shaping expressive performance: a problem-solving approach. *Music Educ. J.* 91:63 10.2307/3400145

[B15] Broomhead P. (2006). A study of instructional strategies for teaching expressive performance in the choral rehearsal. *Bull. Coun. Res. Music Educ.* 167 7–20. 10.2307/40319286

[B16] Broomhead P. Skidmore J. B. Eggett D. L. Mills M. M. (2012). The effects of a positive mindset trigger word pre-performance routine on the expressive performance of junior high age singers. *J. Res. Music Educ.* 60 62–80. 10.1177/0022429411435363

[B17] Broomhead P. Skidmore J. B. Eggett D. L. Mills M. M. (2018). The effects of a teacher-directed preperformance routine on expressive performance mindset. *Bull. Council Res. Music Educ.* 215 57–74. 10.5406/bulcouresmusedu.215.0057

[B18] Cassirer E. (1955). *The Philosophy of Symbolic Forms (Ralph Manheim transl.).* London: Yale University Press.

[B19] Cespedes-Guevara J. Eerola T. (2018). Music communicates affects, not basic emotions – A constructionist account of attribution of emotional meanings to music. *Front. Psychol.* 9:215. 10.3389/fpsyg.2018.00215 29541041 PMC5836201

[B20] Chaffin R. Imreh G. Lemieux A. F. Chen C. (2003). “Seeing the Big Picture”: piano practice as expert problem solving. *Music Percept.* 20 465–490. 10.1525/mp.2003.20.4.465 33021500

[B21] Chester E. (2008). *An Examination of the Relationship Between Teaching Method and Middle School Instrumentalists’ Performance of Three Expressive Skills.* Doctoral dissertation, University of Maryland, College Park, MD.

[B22] Chew E. (2013). “The tipping point analogy for musical timing,” in *Proceedings of the 2nd International Conference of the Performance Studies Network (PSN2)*, Cambridge.

[B23] Chew E. (2014). Music interaction with others as conveyor of relational intent: a response to Cross (2014). *Psychol. Music* 42 826–838. 10.1177/0305735614549407

[B24] Clarke E. F. (1988). “Generative principles in music performance,” in *Generative Processes in Music: The Psychology of Performance, Improvisation, and Composition*, ed. Sloboda J. A. (New York, NY: Clarendon Press), 1–26.

[B25] Coutinho E. Dibben N. (2013). Psychoacoustic cues to emotion in speech prosody and music. *Cogn. Emot.* 27 658–684. 10.1080/02699931.2012.732559 23057507

[B26] Creech A. (2012). Interpersonal behaviour in one-to-one instrumental lessons: an observational analysis. *Br. J. Music Educ.* 29 387–407. 10.1017/S026505171200006X

[B27] Creech A. Gaunt H. (2018). “The changing face of individual instrumental tuition,” in *Vocal, Instrumental, and Ensemble Learning and Teaching. An Oxford Handbook of Music Education*, Vol. 3 eds McPherson G. E. Welch G. F. (Oxford: Oxford University Press), 145–164.

[B28] Cross I. (2005). “Music and meaning, ambiguity and evolution,” in *Musical Communication*, eds Miell D. Macdonald R. Hargreaves D. J. (Oxford: Oxford University Press), 27–43. 10.1093/acprof:oso/9780198529361.003.0002

[B29] Custodero L. A. (2002). Seeking challenge, finding skill: flow experience and music education. *Arts Educ. Policy Rev.* 103 3–9. 10.1080/10632910209600288

[B30] Dahl S. Friberg A. (2007). Visual perception of expressiveness in musicians’ body movements. *Music Percept.* 24 433–454. 10.1525/MP.2007.24.5.433 33021500

[B31] Dalaire M. (2020). *Investigating the Link Between Dalcroze Eurhythmics and Musical Expressivity in Novice Piano Students.* Doctoral dissertation, Université d’Ottawa/University of Ottawa, Ottawa, ON.

[B32] Davidson J. Pitts S. Correia J. S. (2001). Reconciling technical and expressive elements in musical instrument teaching: working with children. *J. Aesth. Educ.* 35 51–62. 10.2307/3333609

[B33] Davidson J. W. (1993). Visual perception of performance manner in the movements of solo musicians. *Psychol. Music* 21 103–113. 10.1177/030573569302100201

[B34] Davidson J. W. (2005). “Bodily communication in musical performance,” in *Music Communication*, eds Miell R. Macdonald D. J. Hargreaves D. (Oxford: Oxford University Press), 215–237. 10.1093/acprof:oso/9780198529361.003.0010

[B35] Davidson J. W. (2018). “The role of bodily movement in learning and performing music: applications for education,” in *Vocal, Instrumental, and Ensemble Learning and Teaching. An Oxford Handbook of Music Education*, Vol. 3 eds McPherson G. E. Welch G. F. (Oxford: Oxford University Press), 226–247.

[B36] Davidson J. W. Correia J. S. (2002). “Body movement,” in *The Science and Psychology of Music Performance: Creative Strategies for Teaching and Learning*, eds Parncutt R. McPherson G. E. (Oxford: Oxford University Press), 237–250.

[B37] DeBellis M. (2005). “Music,” in *The Routledge Companion to Aesthetics*, 2nd Edn, eds Gaut B. N. Lopes D. M. (London: Routledge), 669–682.

[B38] Delalande F. Cornara S. (2010). Music education research sound explorations from the ages of 10 to 37 months: the ontogenesis of musical conducts. *Music Educ. Res.* 12 257–268. 10.1080/14613808.2010.504812

[B39] Dickey M. R. (1992). A review of research on modeling in music teaching and learning. *Bull. Coun. Res. Music Educ.* 113 27–40.

[B40] Doǧantan-Dack M. (2014). “Philosophical reflections on expressive music performance,” in *Expressiveness in Music Performance: Empirical Approaches Across Styles and Cultures*, eds Fabian D. Timmers R. Schubert E. (Oxford: Oxford University Press), 3–21.

[B41] Dowling W. J. (1973). Rhythmic groups and subjective chunks in memory for melodies. *Percep. Psychophys.* 14 37–40. 10.3758/BF03198614

[B42] Ebie B. D. (2004). The effects of verbal, vocally modeled, kinesthetic, and audio-visual treatment conditions on male and female middle-school vocal music students’ abilities to expressively sing melodies. *Psychol. Music* 32 405–417. 10.1177/0305735604046098

[B43] Epperson G. (2016). *Music.* Available online at: https://www.britannica.com/art/music (accessed April 6, 2018).

[B44] Ericsson K. Krampe R. Tesch-Römer C. (1993). The role of deliberate practice in the acquisition of expert performance. *Psychol. Rev.* 100:363 10.1037/0033-295x.100.3.363

[B45] Fabian D. Schubert E. (2009). Baroque expressiveness and stylishness in three recordings of the D minor Sarabanda for solo violin (BWV 1004). *Music Perform. Res.* 3 36–56.

[B46] Fabian D. Timmers R. Schubert E. (Eds). (2014). *Expressiveness in Music Performance: Empirical Approaches Across Styles and Cultures.* Oxford: Oxford University Press 10.1093/acprof:oso/9780199659647.001.0001

[B47] Fortuna S. Nijs L. (2020a). Children’s representational strategies based on verbal versus bodily interactions with music: an intervention-based study. *Music Educ. Res.* 22 107–127. 10.1080/14613808.2019.1699521

[B48] Fortuna S. Nijs L. (2020b). Children’s verbal explanations of their visual representation of the music. *Int. J. Music Educ.* 38 563–581. 10.1177/0255761420932689

[B49] Freire P. (1993). *Pedagogy of the Oppressed.* trans. M. B. Ramos. New York, NY: Continuum.

[B50] Friberg A. Battel G. U. (2002). “Structural communication,” in *The Science and Psychology of Music Performance: Creative Strategies for Teaching and Learning*, eds Parncutt G. E. McPherson R. (Oxford: Oxford University Press), 199–218.

[B51] Friberg A. Sundberg J. (1999). Does music performance allude to locomotion? A model of final ritardandi derived from measurements of stopping runners. *J. Acoust. Soc. Am.* 105 1469–1484. 10.1121/1.426687

[B52] Gabrielsson A. (1999). “Music performance,” in *The Psychology of Music*, 2nd Edn, ed. Deutsch D. (San Diego, CA: Academic Press), 502–602.

[B53] Gabrielsson A. (2001). Emotion perceived and emotion felt: same or different? *Music. Sci.* 5(Suppl. 1) 123–147. 10.1177/10298649020050S105

[B54] Gabrielsson A. Juslin P. N. (1996). Emotional expression in music performance: between the performer’s intention and the listener’s experience. *Psychol. Music* 24 68–91. 10.1177/0305735696241007

[B55] Gabrielsson A. Lindström E. (2010). “The role of structure in the musical expression of emotions,” in *Handbook of Music and Emotion: Theory, Research, Applications*, eds Juslin P. N. Sloboda J. A. (Oxford: Oxford University Press), 367–400. 10.1093/acprof:oso/9780199230143.003.0014

[B56] Gibbs B. K. (2015). Musical expression on wind instruments: perspectives from a panel of experts. *GSTF J. Music (JMusic)* 2 1–10.

[B57] Gilman B. I. (1892a). Report of an experimental test of musical expressiveness. *Am. J. Psychol.* 4 558–576. 10.2307/1410803

[B58] Gilman B. I. (1892b). Report of an experimental test of musical expressiveness (continued). *Am. J. Psychol.* 5 42–73. 10.2307/1410813

[B59] Graham D. (1998). Teaching for creativity in music performance. *Music Educat. J.* 84 24–28. 10.2307/3399126

[B60] Griffiths M. H. (2017). *Fostering the Development of Expressive Performance Skills: A Gestural Approach Within the Reflective, One-to-One Piano Studio.* Doctoral thesis, Griffith University, Mount Gravatt, QLD.

[B61] Háden G. P. Honing H. Török M. Winkler I. (2015). Detecting the temporal structure of sound sequences in newborn infants. *Int. J. Psychophysiol.* 96 23–28. 10.1016/j.ijpsycho.2015.02.024 25722025

[B62] Hallam S. (1998). *Instrumental Teaching: A Practical Guide to Better Teaching and Learning.* London: Heinemann Educational.

[B63] Hallam S. (2006). *Music Psychology in Education.* London: Institute of Education, University of London.

[B64] Hallam S. (2010). “Music education: the role of affect,” in *Handbook of Music and Emotion: Theory, Research, Applications*, eds Juslin J. A. Patrik N. S. (Oxford: Oxford University Press), 791–817.

[B65] Hallam S. Rinta T. Varvarigou M. Creech A. Papageorgi I. Gomes T. (2012). The development of practising strategies in young people. *Psychol. Music* 40 652–680. 10.1177/0305735612443868

[B66] Hawkes M. E. (2018). *The Practical Application of Psychological Skills Training for Musicians: An Exploratory Multi-Method Study.* Doctoral dissertation, The University of Sheffield, Sheffield.

[B67] Hoeschele M. Merchant H. Kikuchi Y. Hattori Y. Ten Cate C. (2015). Searching for the origins of musicality across species. *Philos. Transact. R. Soc. B* 370:20140094. 10.1098/rstb.2014.0094 PMC4321135 25646517

[B68] Honing H. (2009). *Iedereen is Muzikaal.* Amsterdam: Nieuw Amsterdam.

[B69] Honing H. (2012). Without it no music: beat induction as a fundamental musical trait. *Ann. N. Y. Acad. Sci.* 1252 85–91. 10.1111/j.1749-6632.2011.06402.x 22524344

[B70] Honing H. (2018). On the biological basis of musicality. *Ann. N. Y. Acad. Sci.* 1423 51–56. 10.1111/nyas.13638 29542134

[B71] Howat R. (1995). “What do we perform?,” in *The Practice of Performance: Studies in Musical Interpretation* (3-20), ed. Rink J. (Cambridge: Cambridge University Press), 10.1017/CBO9780511552366.002

[B72] Johnson M. D. (1993). Dalcroze skills for all teachers. *Music Educat. J.* 79 42–45. 10.2307/3398597

[B73] Johnson P. (2004). “‘Expressive Intonation’ in string performance: problems of analysis and interpretation,” in *The Music Practitioner: Research for the Music Performer, Teacher and Listener*, ed. Davidson J. W. (Aldershot: Ashgate Publishing Limited), 79–100. 10.4324/9781315085807-7

[B74] Juntunen M. L. Hyvönen L. (2004). The Dalcroze approach: experiencing and knowing music through embodied exploration. *Br. J. Music Educat.* 21 199–214. 10.1017/S0265051704005686

[B75] Juslin P. Laukka P. (2003). Communication of emotions in vocal expressions and music performance: different channel, same code? *Psychol. Bullet.* 129 770–814. 10.1037/0033-2909.129.5.770 12956543

[B76] Juslin P. N. (2000). Cue utilization in communication of emotion in music performance: Relating performance to perception. *J. Exp. Psychol.* 26:1797. 10.1037/0096-1523.26.6.1797 11129375

[B77] Juslin P. N. (2003). Five facets of musical expression: a psychologist’s perspective on music performance. *Psychol. Music* 31 273–302. 10.1177/03057356030313003

[B78] Juslin P. N. Friberg A. Schoonderwaldt E. Karlsson J. (2004). “Feedback learning of musical expressivity,” in *Musical Excellence: Strategies and Techniques to Enhance Performance*, ed. Williamon A. (Oxford: Oxford University Press), 247–270. 10.1093/acprof:oso/9780198525356.003.0013

[B79] Juslin P. N. Karlsson J. Lindström E. Friberg A. Schoonderwaldt E. (2006). Play it again with feeling: computer feedback in musical communication of emotions. *J. Exp. Psychol.* 12 79–95. 10.1037/1076-898X.12.2.79 16802890

[B80] Juslin P. N. Laukka P. (2000). Improving emotional communication in music performance through cognitive feedback. *Music. Sci.* 4 151–183. 10.1177/102986490000400202

[B81] Juslin P. N. Persson R. S. (2002). *Emotional Communication. In The Science & Psychology of Music Performance: Creative Strategies for Teaching and Learning.* Oxford: Oxford University Press, 220–236.

[B82] Juslin P. N. Timmers R. (2010). “Expression and communication of emotion in music performance,” in *Handbook of Music and Emotion: Theory, Research, Applications*, eds Juslin J. A. Patrik N. S. (Oxford: Oxford University Press). 453–489. 10.1093/acprof:oso/9780199230143.001.0001

[B83] Karlsson J. Juslin P. N. (2008). Musical expression: an observational study of instrumental teaching. *Psychol. Music* 36 309–334. 10.1177/0305735607086040

[B84] Karlsson J. Liljeström S. Juslin P. N. (2009). Teaching musical expression: effects of production and delivery of feedback by teacher vs. computer on rated feedback quality. *Music Educ. Res.* 11 175–191. 10.1080/14613800902924532

[B85] Kauchak D. Eggen P. (2012). *Learning and Teaching: Research-Based Methods*, 6th Edn London: Pearson.

[B86] Kendall R. A. Carterette E. C. (1990). The communication of musical expression. *Music Percept.* 8 129–163. 10.2307/40285493

[B87] Kenny D. T. Ackermann B. J. (2016). “Optimizing physical and psychological health in performing musicians,” in *The Oxford Handbook of Music Psychology*, 2 Edn, eds Hallam S. Cross I. Thaut M. (Oxford: Oxford University Press), 633–647.

[B88] Kleine Staarman J. Mercer N. (2010). “The guided construction of knowledge: talk between teachers and students,” in *International Handbook of Psychology in Education*, eds Karen J. L. Clare W. Staarman K. (Bingley: Emerald Group Publishing Limited), 75–104.

[B89] Langer S. K. (1957). *Philosophy in a New Key*, 3 Edn Cambridge, MA: Mentor Books and Harvard University Press.

[B90] Leech-Wilkinson D. Prior H. M. (2014). “Heuristics for expressive performance,” in *Expressiveness in Music Performance: Empirical Approaches Across Styles and Cultures*, eds Fabian D. Timmers R. Schubert E. (Oxford: Oxford University Press), 34–57. 10.1093/acprof:oso/9780199659647.003.0003

[B91] Lehmann A. C. Sloboda J. A. Woody R. H. (2007). *Psychology for Musicians: Understanding and Acquiring the Skills.* Oxford: Oxford University Press.

[B92] Leman M. (2010). An embodied approach to music semantics. *Music. Sci.* 14(Suppl. 1) 43–67. 10.1177/10298649100140S104

[B93] Leman M. (2017). “The interactive dialectics of musical meaning formation,” in *The Routledge Companion to Embodied Music Interaction*, Lesaffre M. Maes P. -J. Leman M. (Abingdon: Taylor & Francis), 13–21. 10.4324/9781315621364-2

[B94] Leman M. Lesaffre M. Maes P.-J. (2017). “Introduction,” in *The Routledge Companion to Embodied Music Interaction*, eds Lesaffre M. Maes P.-J. Leman M. (Abingdon: Taylor & Francis), 1–10. 10.4324/9781315621364-1

[B95] Lesaffre M. Maes P.-J. Leman M. (2017). *The Routledge Companion to Embodied Music Interaction*. (New York, NY: Routledge), 1–10.

[B96] Lindström E. Juslin P. N. Bresin R. Williamon A. (2003). “Expressivity comes from within your soul”: a questionnaire study of music students’ perspectives on expressivity. *Res. Stud. Music Educ.* 20 23–47. 10.1177/1321103X030200010201

[B97] Lisboa T. (2000). *Action and Thought in Cello Playing: An Investigation of Children’s Practice and Performance.* Doctoral dissertation, The University of Sheffield, Sheffield.

[B98] Lisboa T. (2008). Action and thought in cello playing: an investigation of children’s practice and performance. *Int. J. Music Educ.* 26 243–267. 10.1177/0255761408092526

[B99] MacRitchie J. Buck B. Bailey N. J. (2013). Inferring musical structure through bodily gestures. *Music. Sci.* 17 86–108. 10.1177/1029864912467632

[B100] McPhee E. A. (2011). Finding the muse: teaching musical expression to adolescents in the one-to-one studio environment. *Int. J. Music Educ.* 29 333–346. 10.1177/0255761411421084

[B101] McPherson G. E. Davidson J. W. Faulkner R. (2012). *Music in Our Lives: Rethinking Musical Ability, Development, and Identity.* New York, NY: Oxford University Press 10.1093/acprof:oso/9780199579297.001.0001

[B102] McPherson G. E. Hallam S. (2016). “Musical potential,” in *The Oxford Handbook of Music Psychology*, 2 Edn, eds Hallam S. Cross I. Thaut M. (Oxford: Oxford University Press), 433–448.

[B103] McPherson G. E. Renwick J. M. (2001). A Longitudinal study of self-regulation in children’s musical practice. *Music Educ. Res.* 3 169–186. 10.1080/14613800120089232

[B104] McPherson G. E. Renwick J. M. (2010). A longitudinal study of self- regulation in children’s musical practice A longitudinal study of self-regulation in children’s musical practice. *Music Educ. Res.* 3 37–41. 10.1080/1461380012008923

[B105] Meissner H. (2017). Instrumental teachers’ instructional strategies for facilitating children’s learning of expressive music performance: an exploratory study. *Int. J. Music Educ.* 35 118–135. 10.1177/0255761416643850

[B106] Meissner H. (2018). *Teaching Young Musicians Expressive Performance: A Mixed Methods Study*. Doctoral dissertation, University of Sheffield, Sheffield.

[B107] Meissner H. (2020). “Children’s views on their learning of performance expression in an action research project,” in *Make Music Matter – Music Education Meeting the Needs of Young Learners*, eds Houmann A. Saether E. (Helbling: European Association for Music in Schools), 153–169.

[B108] Meissner H. Timmers R. (2019). Teaching young musicians expressive performance: an experimental study. *Music Educ. Res.* 21 20–39. 10.1080/14613808.2018.1465031

[B109] Meissner H. Timmers R. (2020). Young musicians’ learning of expressive performance: the importance of dialogic teaching and modeling. *Front. Psychol.* 5:11 10.3389/feduc.2020.00011 PMC7830251 33505334

[B110] Meissner H. Timmers R. Pitts S. (2019). “Just notes”: young musicians’ perspectives on learning expressive performance. *Res. Stud. Music Educ.* 11 652–680. 10.1177/1321103X19899171

[B111] Meissner H. Timmers R. Pitts S. (2020). “Just notes”: young musicians’ perspectives on learning expressive performance. *Res. Stud. Music Educ.* 11 652–680. 10.1177/1321103X19899171

[B112] Mercer N. (1995). *The Guided Construction of Knowledge: Talk Amongst Teachers and Learners.* Clevedon: Multilingual Matters.

[B113] Moog H. (1976). *The Musical Experience of the Pre-School Child.* trans. C. Clarke. London: Schott Music Corp.

[B114] Moorhead G. E. Pond D. (1942). *Music of Young Children, II. General Observations.* Santa Barbara, CA: Pillsbury Foundation for Advancement of Music Education.

[B115] Mornell A. Wulf G. (2019). Adopting an external focus of attention enhances musical performance. *J. Res. Music Educ.* 66 375–391. 10.1177/0022429418801573

[B116] Nijs L. (2018). Dalcroze meets technology: integrating music, movement and visuals with the music paint machine. *Music Educ. Res.* 20 163–183. 10.1080/14613808.2017.1312323

[B117] Nusseck M. Wanderley M. M. (2009). Music and motion——how music-related ancillary body movements contribute to the experience of music. *Music Percept.* 26 335–353. 10.1525/mp.2009.26.4.335 33021500

[B118] Ockelford A. (2000). Music in the education of children with severe or profound learning difficulties: issues in current UK provision, a new conceptual framework, and proposals for research. *Psychol. Music* 28 197–217. 10.1177/0305735600282009

[B119] Overy K. Nicolson R. I. Fawcett A. J. Clarke E. F. (2003). Dyslexia and music: measuring musical timing skills. *Dyslexia* 9 18–36. 10.1002/dys.233 12625374

[B120] Owens P. Sweller J. (2008). Cognitive load theory and music instruction. *Educ. Psychol.* 28 29–45. 10.1080/01443410701369146

[B121] Palmer C. (1996). On the assignment of structure in music performance. *Music Percept.* 14 23–56. 10.2307/40285708

[B122] Palmer C. (1997). Music performance. *Annu. Rev. Psychol.* 48 115–138. 10.1146/annurev.psych.48.1.115 9046557

[B123] Papageorgi I. Kopiez R. (2018). “Psychological and Physiological aspects of learning to perform,” in *Vocal, Instrumental, and Ensemble Learning and Teaching. An Oxford Handbook of Music Education*, Vol. 3 eds McPherson G. E. Welch G. F. (Oxford: Oxford University Press), 184–208.

[B124] Persson R. (1996). Brilliant performers as teachers: a case study of commonsense teaching in a conservatoire setting. *Int. J. Music Educ.* 1 25–36. 10.1177/025576149602800103

[B125] Pike P. D. (2017). Self-regulation of teenaged pianists during at-home practice. *Psychol. Music* 45 739–751. 10.1177/0305735617690245

[B126] Pitts S. (2005). *Valuing Musical Participation.* Aldershot: Ashgate Publishing Limited.

[B127] Pitts S. Davidson J. (2000). Developing effective practise strategies: Case studies of three young instrumentalists. *Music Educ. Res*. 2 45–56. 10.1080/14613800050004422

[B128] Pitts S. E. Davidson J. W. McPherson G. E. (2000). Models of success and failure in instrumental learning: case studies of young players in the first 20 months of learning. *Bull. Council Res. Music Educ.* 146 51–69. 10.2307/40319033

[B129] Repp B. H. (1992). Diversity and commonality in music performance: an analysis of timing microstructure in Schumann’s “‘Träumerei.”’. *J. Acoust. Soc. Am.* 92 2546–2568. 10.1121/1.404425 1479119

[B130] Repp B. H. (1998). A microcosm of musical expression. I. Quantitative analysis of pianists’ timing in the initial measures of Chopin’s Etude in E major. *J. Acoust. Soc. Am.* 104 1085–1100. 10.1121/1.423325 9714927

[B131] Rosenblatt L. (1938). *Literature as Exploration.* New York, NY: Modern Language Association of America.

[B132] Rosenthal R. K. (1984). The relative effects of guided model, model only, guide only, and practice only treatments on the accuracy of advanced instrumentalists’ musical performance. *J. Res. Music Educ.* 32 265–273. 10.2307/3344924

[B133] Rostvall A.-L. West T. (2003). Analysis of interaction and learning in instrumental teaching. *Music Educ. Res.* 5 213–226. 10.1080/1461380032000126319

[B134] Rostvall A.-L. West T. (2005). Theoretical and methodological perspectives on designing video studies of interaction. *Int. J. Qual. Methods* 4 87–108. 10.1177/160940690500400406

[B135] Sang R. C. (1987). A study of the relationship between instrumental music teachers’ modeling skills and pupil performance behaviors. *Bullet. Council Res. Music Educ.* 91 155–159.

[B136] Schellenberg E. Krysciak A. Campbell R. (2000). Perceiving emotion in melody: Interactive effects of pitch and rhythm. *Music Percept.* 18 155–171. 10.2307/40285907

[B137] Schiavio A. (2019). “The primacy of experience. phenomenology, embodiment, and assessments in music education,” in *The Oxford Handbook of Philosophical and Qualitative Perspectives on Assessment in Music Education*, eds Elliott D. J. Silverman M. McPherson G. (New York, NY: Oxford University Press), 65–82.

[B138] Schiavio A. van der Schyff D. (2018). 4E Music pedagogy and the principles of self-organization. *Behav. Sci.* 8:72. 10.3390/bs8080072 30096864 PMC6115738

[B139] Schiavio A. van der Schyff D. Biasutti M. Moran N. Parncutt R. (2019). Instrumental technique, expressivity, and communication. A qualitative study on learning music in individual and collective settings. *Front. Psychol.* 10:737. 10.3389/fpsyg.2019.00737 31001179 PMC6457278

[B140] Schiavio A. van der Schyff D. Kruse-Weber S. Timmers R. (2017). When the sound becomes the goal. 4E cognition and teleomusicality in early infancy. *Front. Psychol.* 8:1585. 10.3389/fpsyg.2017.01585 28993745 PMC5622185

[B141] Schippers H. (2006). ‘As if a little bird is sitting on your finger.’: metaphor as a key instrument in training professional musicians. *Int. J. Music Educ.* 24 209–217. 10.1177/0255761406069640

[B142] Schubert E. Fabian D. (2014). “A taxonomy of listeners’ judgements of expressiveness in music performance,” in *Expressiveness in Music Performance: Empirical Approaches Across Styles and Cultures*, eds Fabian D. Timmers R. Schubert E. (Oxford: Oxford University Press), 283–303. 10.1093/acprof:oso/9780199659647.003.0016

[B143] Seashore C. E. (1923). Measurements on the expression of emotion in music. *Proc. Natl. Acad. Sci.* 9 323–325. 10.1073/pnas.9.9.323 16576728 PMC1085412

[B144] Seitz J. A. (2005). Dalcroze, the body, movement and musicality. *Psychol. Music* 33 419–435. 10.1177/0305735605056155

[B145] Shaffer L. H. (1992). “How to interpret music,” in *Cognitive Bases of Musical Communication*, eds Riess Jones M. Holleran S. (Washington, DC: American Psychological Association).

[B146] Shaffer L. H. (1995). Musical performance as interpretation. *Psychol. Music* 23 17–38. 10.1177/0305735695231002

[B147] Silverman M. (2007). Musical interpretation: philosophical and practical issues. *Int. J. Music Educ.* 25 101–117. 10.1177/0255761407079950

[B148] Silverman M. (2020). Sense-making, meaningfulness, and instrumental music education. *Front. Psychol.* 11:837. 10.3389/fpsyg.2020.00837 32435220 PMC7219105

[B149] Skidmore D. Murakami K. (2016). *Dialogic Pedagogy: The Importance of Dialogue in Teaching and Learning.* Bristol: Multilingual Matters.

[B150] Sloboda J. (1996). “The acquisition of musical performance expertise: deconstructing the ‘talent’ account of individual differences in musical expressivity,” in *The Road to Excellence: The Acquisition of Expert Performance in the Arts and Sciences, Sport and Games*, ed. Ericsson K. A. (Mahwah, NJ: Lawrence Erlbaum Associates), 107–126.

[B151] Sloboda J. Davidson J. (1996). “The young performing musician,” in *Musical Beginnings: Origins and Development of Musical Competence*, eds Deliège I. Sloboda J. (Oxford: Oxford University Press), 171–190. 10.1093/acprof:oso/9780198523321.003.0007

[B152] Sloboda J. A. (2005). *Exploring the Musical Mind: Cognition, Emotion, Ability, Function.* Oxford: Oxford University Press 10.1093/acprof:oso/9780198530121.001.0001

[B153] Sosniak L. A. (1990). “The tortoise, the hare, and the development of talent,” in *Encouraging the Development of Exceptional Skills and Talents*, ed. Howe M. J. A. (Leicester: British Psychological Society), 149–164.

[B154] Stambaugh L. A. (2013). Differential effects of cognitive load on university wind students’ practice. *Psychol. Music* 41 749–763. 10.1177/0305735612449505

[B155] Swanwick K. (2011). *Teaching Music Musically (Classic Edition).* London: Routledge.

[B156] Sweller J. (1988). Cognitive load during problem solving: effects on learning. *Cogn. Sci.* 12 257–285. 10.1016/0364-0213(88)90023-7

[B157] Tafuri J. (2008). *Infant Musicality: New Research for Educators and Parents*, ed. Welch G., trans. Hawkins E. (Farnham: Ashgate Publishing Limited).

[B158] Tagg P. (2012). *Music’s Meanings: A Modern Musicology for Non-Musos.* New York, NY: Mass Media Music Scholars’ Press.

[B159] Tan Y. T. McPherson G. E. Peretz I. Berkovic S. F. Wilson S. J. (2014). The genetic basis of music ability. *Front. Psychol.* 5:658. 10.3389/fpsyg.2014.00658 25018744 PMC4073543

[B160] Timmers R. (2018). “Music and emotion,” in *Foundations of Music Psychology: Theory and Research*, eds Rentfrow J. Levitin D. J. (Cambridge, MA: MIT Press).

[B161] Timmers R. (2020). Analyzing relationships between color, emotion and music using Bayes’ rule in Bach’s Well-Tempered Clavier Book 1. *Int. J. Music Sci. Technol. Art* 2 40–47.

[B162] Timmers R. Ashley R. (2007). Emotional ornamentation in performances of a Handel sonata. *Music Percept.* 25 117–134. 10.1525/mp.2007.25.2.117 33021500

[B163] Timmers R. Desain P. (2000). “Vibrato: questions and answers from musicians and science,” in *Proceedings of the Sixth International Conference on Music Perception and Cognition*, Vol. 2 Available onlie at: http://cf.hum.uva.nl/mmm/papers/mmm-35/mmm-35.pdf (accessed December 16, 2020).

[B164] Timmers R. Honing H. (2002). On music performance, theories, measurement and diversity. *Cognitive Proces. (Int. Q. Cogn. Sci.)* 31 1–33. 10.1163/ej.9789004184343.i-316.5

[B165] Timmers R. Marolt M. Camurri A. Volpe G. (2006). Listeners’ emotional engagement with performances of a Scriabin étude: an explorative case study. *Psychol. Music* 34 481–510. 10.1177/0305735606067165

[B166] Timmers R. Sadakata M. (2014). “Training expressive performance by means of visual feedback: existing and potential applications of performance measurement techniques,” in *Expressiveness in Music Performance: Empirical Approaches Across Styles and Cultures*, eds Fabian D. Timmers R. Schubert E. (Oxford: Oxford University Press), 304–322. 10.1093/acprof:oso/9780199659647.003.0017

[B167] Todd N. (1985). A model of expressive timing in tonal music. *Music Percept.* 3 33–57. 10.2307/40285321

[B168] Trapkus P. (2020). Teaching musical interpretation: a student-centred model for addressing a fundamental concept. *Am. String Teach.* 70 17–21. 10.1177/0003131319891147

[B169] Trehub S. Hannon E. E. Schachner A. (2010). “Perspectives on music and affect in the early years,” in *Handbook of Music and Emotion: Theory, Research, Applications*, eds Juslin P. N. Sloboda J. A. (Oxford: Oxford University Press), 645–668. 10.1093/acprof:oso/9780199230143.003.0023

[B170] Trehub S. E. (2003). The developmental origins of musicality. *Nat. Neurosci.* 6 669–673. 12830157 10.1038/nn1084

[B171] Trehub S. E. (2016). “Infant musicality,” in *The Oxford Handbook of Music Psychology*, 2nd Edn, eds Hallam S. Cross I. Thaut M. (Oxford: Oxford University Press), 387–397.

[B172] Trehub S. E. Nakata T. (2001). Emotion and music in infancy. *Music. Sci.* 5(Suppl. 1) 37–61. 10.1177/10298649020050S103

[B173] Trevarthen C. (2002). “Origins of musical identity: evidence from infancy for musical social awareness,” in *Musical Identities*, eds Macdonald R. Hargreaves D. Miell D. (Oxford: Oxford University Press), 21–38.

[B174] Van Zijl A. G. Luck G. (2013). “The sound of sadness: the effect of performers’ emotions on audience ratings,” in *Proceedings of the 3rd International Conference on Music & Emotion, Jyväskylä, Finland, June 11-15, 2013*, (Jyväskylä: University of Jyväskylä, Department of Music).

[B175] Van Zijl A. G. W. Sloboda J. (2011). Performers’ experienced emotions in the construction of expressive musical performance: an exploratory investigation. *J. Sagepub. Com.* 39 196–219. 10.1177/0305735610373563

[B176] Van Zijl A. G. W. Toiviainen P. Lartillot O. Luck G. (2014). The sound of emotion: the effect of perfomers’ experienced emotions on auditory performance characeristics. *Music Percept.* 32 33–50. 10.1525/mp.2014.32.1.33 33021500

[B177] Vandewalker D. (2014). *Relative Effectiveness of Three Diverse Instructional Conditions on Seventh-Grade Wind Band Students ’ Expressive Musical Performance.* Boston, MA: Boston University.

[B178] Von Glasersfeld E. (2012). “A constructivist approach to teaching,” in *Constructivism in Education*, eds Steffe L. P. Gale J. (Hillsdale, MI: Erlbaum), 3–15. 10.4324/9780203052600-5

[B179] Vygotsky L. (1986). *Thought and Language.* ed. and trans. A. Kozulin Cambridge, MA: MIT Press.

[B180] Vygotsky L. S. (1978). *Mind in Society: The Development of Higher Psychological Processes*, eds Cole M. John-Steiner V. Scribner S. Souberman E. (Cambridge, MA: Harvard University Press).

[B181] Wanderley M. M. Vines B. (2006). “Origins and functions of clarinettist’s ancillary gestures,” in *Music and Gesture*, eds Gritten A. King E. (Aldershot: Ashgate Publishing Limited), 165–191. 10.4324/9781315091006-11

[B182] Ward V. (2007). Teaching musical awareness: the development and application of a ‘toolkit’ of strategies for instrumental teachers. *Br. J. Music Educ.* 24 21–36. 10.1017/s0265051706007200

[B183] Watt R. J. Ash R. L. (1998). A psychological investigation of meaning in music. *Music. Sci.* 2 33–53. 10.1177/102986499800200103

[B184] West T. Rostvall A.-L. (2003). A Study of interaction and learning in instrumental teaching. *Int. J. Music Educ.* 40 16–27. 10.1177/025576140304000103

[B185] Wiggins J. (2016). “Musical agency,” in *The Child as Musician: A Handbook of Musical Development*, 2nd Edn, ed. McPherson G. E. (Oxford, UK: Oxford University Press), 102–121. 10.1093/acprof:oso/9780198744443.003.0006

[B186] Windsor W. Aarts R. Desain P. Heijink H. Timmers R. (2000). “On time: the influence of tempo, structure and style on the timing of grace notes in skilled musical performance,” in *Rhythm Perception and Production*, eds Desain P. Windsor W. L. (Lisse: Swets & Zeitlinger), 217–223.

[B187] Wood D. Bruner J. S. Ross G. (1976). The role of tutoring in problem solving. *J. Child Psychol. Psychiatry* 17 89–100. 10.1111/j.1469-7610.1976.tb00381.x 932126

[B188] Woody R. H. (2000). Learning expressivity in music performance: an exploratory study. *Res. Stud. Music Educ.* 14 14–23. 10.1177/1321103X0001400102

[B189] Woody R. H. (2001). Learning from the experts: applying research in expert performance to music education. *Update* 19 9–14. 10.1177/87551233010190020103

[B190] Woody R. H. (2003). Explaining expressive performance: component cognitive skills in an aural modeling task. *J. Res. Music Educ.* 51 51–63. 10.2307/3345648

[B191] Woody R. H. (2006a). The effect of various instructional conditions on expressive music performance. *J. Res. Music Educ*. 54 21–36. 10.1177/002242940605400103

[B192] Woody R. H. (2006b). Musicians’ cognitive processing of imagery-based instructions for expressive performance. *J. Res. Music Educ.* 54 125–137. 10.1177/002242940605400204

[B193] Wulf G. (2013). Attentional focus and motor learning: a review of 15 years. *Int. Rev. Sport Exerc. Psychol.* 6 77–104. 10.1080/1750984X.2012.723728

[B194] Wulf G. Guss-West C. Hum B. (2016). Attentional Focus in classical ballet a survey of professional dancers. Ingentaconnect.Com. *J. Dance Med. Sci.* 20 23–29. 10.12678/1089-313X.20.1.23 27025449

[B195] Wulf G. Shea C. Lewthwaite R. (2010). Motor skill learning and performance: a review of influential factors. *Med. Educ.* 44 75–84. 10.1111/j.1365-2923.2009.03421.x 20078758

[B196] Yandell N. (2018). *Performance Improves When Exposed to Audiences – Even Imaginary Ones | Debate | The Strad.* Available online at: https://www.thestrad.com/debate-old/performances-develop-and-improve-through-exposure-to-an-audience–even-an-imaginary-one-/7680.article (accessed December 16, 2020).

